# Glycan diversity in ovarian cancer: Unraveling the immune interplay and therapeutic prospects

**DOI:** 10.1007/s00281-024-01025-6

**Published:** 2024-10-21

**Authors:** Gerrit Wolters-Eisfeld, Leticia Oliveira-Ferrer

**Affiliations:** 1 Department of General, Visceral and Thoracic Surgery, University Medical Center Hamburg-Eppendorf, Hamburg, Germany; 2 Department of Gynecology, University Medical Center Hamburg-Eppendorf, Hamburg, Germany

**Keywords:** Ovarian cancer, Glycosylation, Glycan diversity, Immune interplay, Immunotherapy

## Abstract

Ovarian cancer remains a formidable challenge in oncology due to its late-stage diagnosis and limited treatment options. Recent research has revealed the intricate interplay between glycan diversity and the immune microenvironment within ovarian tumors, shedding new light on potential therapeutic strategies. This review seeks to investigate the complex role of glycans in ovarian cancer and their impact on the immune response. Glycans, complex sugar molecules decorating cell surfaces and secreted proteins, have emerged as key regulators of immune surveillance in ovarian cancer. Aberrant glycosylation patterns can promote immune evasion by shielding tumor cells from immune recognition, enabling disease progression. Conversely, certain glycan structures can modulate the immune response, leading to either antitumor immunity or immune tolerance. Understanding the intricate relationship between glycan diversity and immune interactions in ovarian cancer holds promise for the development of innovative therapeutic approaches. Immunotherapies that target glycan-mediated immune evasion, such as glycan-based vaccines or checkpoint inhibitors, are under investigation. Additionally, glycan profiling may serve as a diagnostic tool for patient stratification and treatment selection. This review underscores the emerging importance of glycan diversity in ovarian cancer, emphasizing the potential for unraveling immune interplay and advancing tailored therapeutic prospects for this devastating disease.

## Introduction

Ovarian cancer is the deadliest of all female cancers, with a poor survival rate at 5 years of approx. 50,8%, which has barely improved in recent decades [1]. Because symptoms are usually vague at early stages, ovarian cancer is often diagnosed at advanced stages, making it difficult to cure [2]. There are three main types of ovarian cancer: epithelial carcinoma, germ cell cancer, and sex-cord-stromal cancer, with the latter two accounting for only approximately 5% of all ovarian cancers [3]. Epithelial ovarian cancer can be further classified into four primary histological subtypes: serous, endometrioid, mucinous and clear cell carcinoma. A more accurate classification on the basis of histological and molecular genetic characteristics assumes two different pathogenesis pathways: "low-grade" type I tumors develop gradually from benign precursors in the ovary via borderline tumors to invasive carcinomas, and "high-grade" type II tumors develop rapidly without detectable precursor lesions from tubal epithelia [4, 5]. While the first type of tumor has characteristic gene alterations according to the specific morphological type, i.e., KRAS and BRAF mutations in serous and mucinous carcinomas and alterations in the β-catenin and PTEN genes in endometroids, type II tumors show marked chromosomal instability, and 50–80% of cases at all stages have p53 mutations, suggesting an early event in tumorigenesis [6].

Despite the relatively well-characterized genetic and biological differences between the aforementioned subtypes, no type-specific therapeutic targets have been identified thus far. The usual treatment is surgical removal to reach no residual disease and platinum-based chemotherapy supplemented with antiangiogenic agents [7]. A major improvement in maintenance therapy has been achieved via the use of inhibitors against poly (ADP‒ribose) polymerase (PARP) molecules, which are involved in the DNA damage repair process. Although the majority of ovarian cancer patients initially respond well to current treatments, 70% experience relapse, underscoring the critical need for novel therapeutic strategies. Despite the promise of immuno-oncological treatments for ovarian cancer, as evidenced by high concentrations of tumor-infiltrating lymphocytes significantly correlating with improved survival rates [8], initial clinical studies have yielded disappointing results. Very modest single-agent activity of various antibodies targeting programmed cell death protein 1 (PD-1) or its ligand PD-L1, with response rates ranging from 4 to 15%, has been reported [9]. Several reasons for this low response rate have been suggested, i.e., low expression levels of immune checkpoint molecules on ovarian cancer cells as well as a low tumor mutational burden, which was recently described as an effective predictive biomarker of the response to immunotherapy [10, 11]. However, a significant gap remains in our understanding of the molecular processes governing tumour-immune interactions. Within this framework, glycosylation is garnering increasing attention due to its relevance, given that the majority of membrane proteins, including immune checkpoint molecules, are glycoproteins, and differential glycosylation could exert a profound effect on the modulation of the immune response in cancer.

**Fig. 1:**
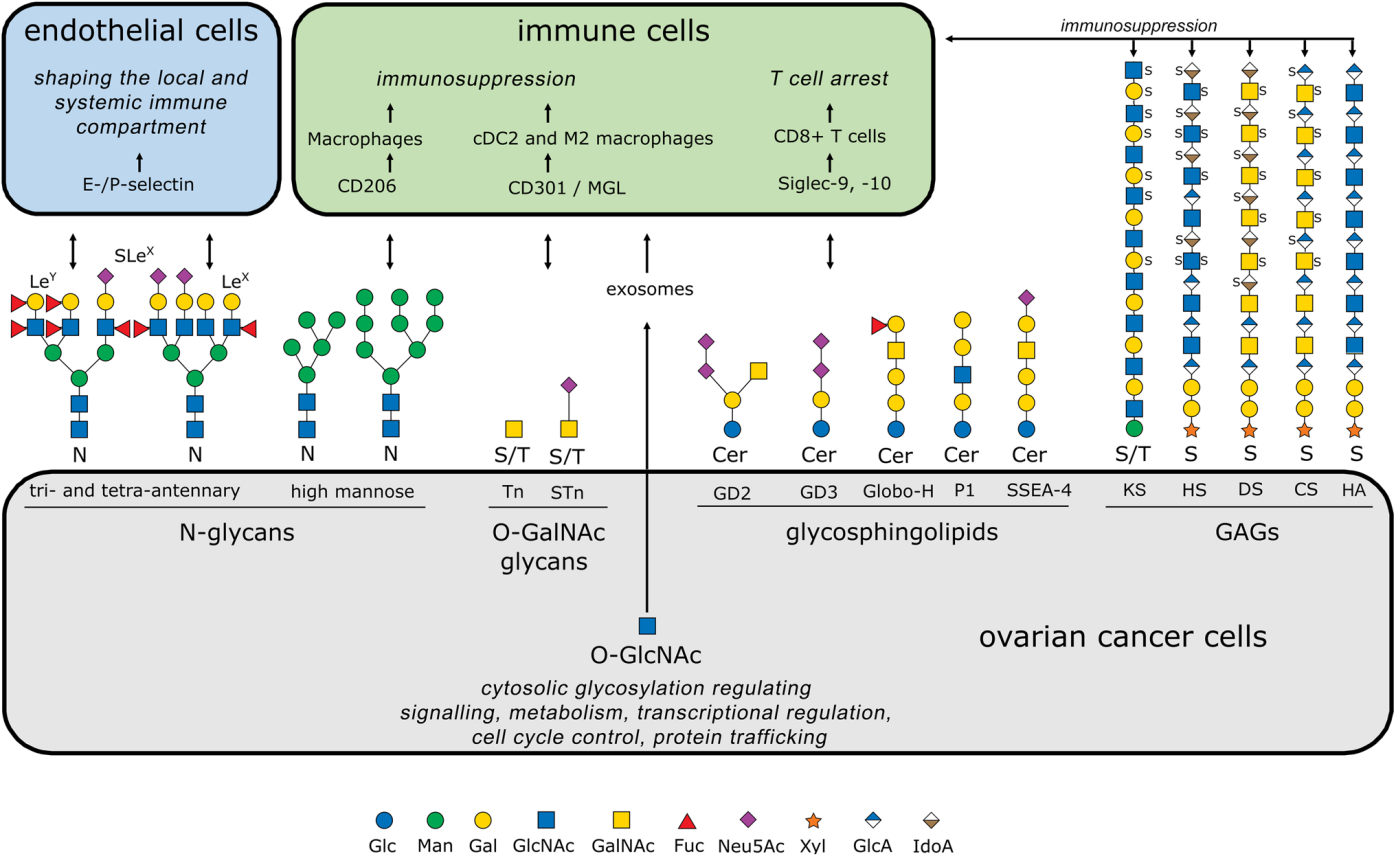
Schematic representation of aberrant glycan structures expressed in ovarian cancer cells (gray box) in modified IUPAC-condensed nomenclature via the web application GlycoGlyph [167]. The corresponding glycan-binding receptors on the endothelium (blue) and immune cells (green) are depicted, along with the cellular effects they mediate. Double-headed arrows indicate interactions between glycans and glycoreceptors. Terminal Lewis structures on N-glycans interact with E- and P-selectins on endothelial cells, playing a crucial role in shaping both local and systemic immune responses. High-mannose glycans are recognized by CD206 on macrophages and, like O-glycosidic Tn and STn antigens, contribute to an immunosuppressive environment through engagement of CD301 on CDC2 and M2 macrophages. In ovarian carcinoma, tumor-specific glycosphingolipids induce T-cell arrest by interacting with Siglec-9 and Siglec-10 on CD8+ T cells, further promoting immune evasion. Additionally, glycosaminoglycans (GAGs) expressed in ovarian carcinoma, such as keratan sulfate (KS-III), contribute to immunosuppression. The heterogeneous sulfation patterns of these GAGs serve as further examples of their complexity. Cytosolic proteins from ovarian carcinoma cells, modified by O-GlcNAc, can be transported to the tumor microenvironment via exosomes and subsequently internalized by immune cells. This mechanism supports immune evasion and further undermines the host immune response against the tumor. *Fuc,* fucose; *Gal,* galactose; *GalNAc,* N-acetylgalactosamine; *Glc,* glucose; *GlcA,* glucuronic acid; *GlcNAc,* N-acetylglucosamine; *IdoA,* iduronic acid; *Man,* mannose; *Neu5Ac,* N-acetylneuraminic acid (sialic acid); *Xyl*, xylose; *S,* sulfate group; *KS*, keratan sulfate; *HS,* heparan sulfate; *DS,* dermatan sulfate; *CS,* chondroitin sulfate; *HA,* hyaluronic acid; *Cer*, ceramide

Glycosylation, the most prevalent and intricate posttranslational modification, plays a significant role in expanding an organism's proteome beyond what is encoded by the genome. Furthermore, it has profound effects on various cellular processes, including cell growth, differentiation, transformation, adhesion, and immune surveillance against tumors [12, 13]. Cell surface glycans and glycolipids constitute the major portion of the membrane (glycocalyx) and secreted and proteolytically shed molecules from all cell types. The major types of cell surface glycans include N-linked glycans, O-linked glycans, glycosaminoglycans (GAGs) and glycosphingolipids (Fig. 1). The two primary forms of glycosylation, N-glycosylation and mucin-type O-glycosylation, involve a series of enzymatic reactions occurring in the endoplasmic reticulum (ER) and Golgi complex. Abnormal glycosylation has been implicated in a wide range of human diseases, including autoimmune disorders [14], infections caused by bacteria and viruses [15], parasites [16], and, notably, cancer [17]. Owing to the intricate nature of protein glycosylation and its profound influence on various biological processes, it is unsurprising that even minor modifications in carbohydrate structure can have a substantial effect on cellular biology. Considering that neoplastic transformation involves alterations in cellular behavior and modifications in protein glycosylation, achieving a thorough understanding of the mechanisms and implications of glycosylation changes associated with neoplastic disease is crucial [18]. Although there has been considerable interest in their role in cancer aggressiveness, the interaction between glycosylation and the immune system has largely been neglected. However, there is a consensus that changes in glycosylation profoundly influence how tumors are recognized by the immune system and can trigger immunosuppressive signaling by interacting with glycan-binding proteins [19].

In this review, the primary emphasis is placed on studies examining alterations in glycoprotein glycans, specifically in ovarian cancer. We explore the effects of these modifications on the progression of neoplastic disease, particularly in relation to the glycan-mediated immune response. Furthermore, we illuminate potential avenues for utilizing these changes as diagnostic indicators and targets for therapeutic interventions.

## Aberrant glycosylation in ovarian *cancer*

Research in cancer glycobiology provides evidence that the glycome present on the surface of cancer cells, released extracellular vesicles, and secreted molecules undergo fundamental changes [20]. Overall, the altered glycosylation observed in cancer cells, including those arising from the ovaries or fallopian tubes, is a complex phenomenon influenced by multiple factors, including dysregulated glycosyltransferase activity, changes in the tumor microenvironment, inflammation, and the generation of tumor-specific glycan structures [17]. These alterations give rise to cancer-associated glycosignatures that are distinguishable from those found in healthy cells [18]. In this context, an ongoing observational study is prospectively recruiting deidentified blood samples and data from women with known pelvic masses to validate ovarian cancer-specific glycosignatures and further distinguish benign from malignant diagnoses. The underlying technology of this liquid biopsy-based trial combines mass spectrometry and artificial intelligence/machine learning to detect tumor-associated changes in circulating glycoproteins (NCT03837327).

In this section, we review and describe recent findings on the role of altered glycosylation in ovarian cancer, with a focus on N-glycans, O-glycans, glycosphingolipids and glycosaminoglycans, as depicted in Fig. 1.

### N-glycosylation

In the process of N-glycosylation, an oligosaccharide precursor (Glc_3_Man_9_GlcNAc_2_-P-P-Dolichol) is initially synthesized in the ER through the collaborative action of enzymes from the asparagine-linked glycosylation (ALG) and dolichol-phosphate mannosyltransferase (DPM) families. This precursor is tethered to a lipid (dolichol) and serves as the foundation for N-glycan assembly. This lipid-linked precursor is subsequently cotranslationally transferred to asparagine (Asn/N) residues within newly synthesized proteins. This transfer is facilitated by an oligosaccharyltransferase (OST) complex, with ribophorin (RPN1) serving as the catalytic subunit [21].

The N-glycan, which is now attached to the protein, undergoes a series of sequential modifications. Enzymes such as glucosidases (GCS1, GANAB) and mannosidases (including MAN1A1) collaboratively trim the glycan structure. Moreover, glycosyltransferases, notably mannosyl-glycoprotein N-acetylglucosaminyltransferases (e.g., MGAT1), add further modifications. This complex process results in a diverse array of N-glycans, which can be categorized into various types, including high-mannose, complex, or hybrid structures [22].

Alterations in N-glycan structures and deregulation of associated glycosylation enzymes in ovarian cancer have been extensively described in recent decades [23, 24]. More specifically, the significance for diagnosis, prognosis, and treatment response has been assessed by analyzing different body fluids, such as serum, tumor tissue, and ascites [25]. Reports on the effects of altered N-glycosylation on tumor cell function have further supported the central importance of this specific posttranslational modification in the pathogenesis and progression of ovarian cancer [23]. In this context, the N-glycan-mediated modulation of tumor-immune cell interactions has gained particular interest.

As mentioned earlier, one of the reasons for the high mortality rate in patients with ovarian cancer is the lack of sensitive diagnostic markers at early stages. In this context, using MALDI-TOF–MS, Biskup and colleagues developed a serum N-glycan score (GLYCOV) capable of differentiating between early-stage ovarian cancer patients and healthy volunteers, with a sensitivity of 95%, which was 35% higher than that of CA125. Notably, the GLYCOV exhibited superior performance (95% sensitivity and 80% specificity) compared with CA125 (60% sensitivity and 65% specificity) in distinguishing early-stage epithelial ovarian cancer patients from benign ovarian disease patients. The GLYCOV score was calculated from the relative areas of 11 N-glycan biomarkers, namely, four high-mannose and seven complex-type fucosylated N-glycans. Here, the authors hypothesize that even at early stages, cytokines produced by ovarian cancer cells induce the production of acute phase proteins released by hepatocytes and modulate their N-glycosylation pattern [26]. Similarly, increased levels of tri- and tetra-antennary oligosaccharides structures were found in the serum of ovarian cancer patients compared with healthy donors, and this trend was often enhanced in follow-up samples during treatment and disease progression [27]. Identical modulation of the N-glycome was found in ascitic fluid from patients with advanced ovarian cancer, although quantitatively, the antennal, sialylation, and fucosylation of the outer antennae were reduced, likely due to differences in protein composition between the two fluids [28].

Modulation of N-glycans has also been described in tumor tissue samples from ovarian cancer patients. Regiospecific N-glycan sialylation has been observed in both the tumor and tumor-stroma tissues of ovarian cancer patients, as well as in tumors with low malignant potential, such as borderline tumors [29]. Here, higher expression of high-mannose glycans and lower expression of hybrid-type glycans were found in epithelial ovarian cancer samples than in healthy controls [30]. Distinct N-glycan structures were identified in association with specific stages and molecular subtypes [31]. Notably, the 'differentiated', 'stromal',' and 'mesenchymal' subtypes were enriched in sialylated and/or fucosylated intact glycopeptides, whereas the 'immunoreactive' and 'proliferative' subtypes presented elevated levels of intact glycopeptides characterized by a high mannose content [32]. In addition, within heterospheroids, ovarian cancer stem cells (CSCs) are responsible for driving the increased expression of the M2 macrophage marker CD206 compared with that in bulk ovarian cancer cells. CD206 is a C-type lectin that binds high-mannose glycans, implying a fundamentally more immunosuppressive program. Moreover, a more sustained elevation in aldehyde dehydrogenase (ALDH) activity within heterospheroids that harbor prepolarized CD206 positive M2 macrophages was noted, indicating a mutually reinforcing interaction that fuels both protumoral activation and self-renewal of CSCs [33].

In line with these findings, an oncogenic role of the α-mannosidase MAN1A1 has been described in ovarian cancer. Here, high MAN1A1 levels in tumors were associated with shorter overall survival (OS) and recurrence-free survival (RFS) in ovarian cancer patients. Mechanistically, reduced MAN1A1 activity alters the ability of tumor cells to aggregate during peritoneal dissemination by modulating the function of various adhesion molecules, such as activated leukocyte cell adhesion molecule (ALCAM) [34]. Modulation of the N-glycome in ovarian cancer cell lines [35, 36] and its effects on cellular properties and functions such as cell adhesion, survival, epithelial–mesenchymal transition, and chemoresistance [37–42] have been studied in detail in vitro. The latter is of clinical relevance, as a panel of three elevated glycan structures (Lewis-type biantennary glycan, Lewis-type triantennary trisialylated glycan) in combination with ovarian cancer-related tumor marker 125 (CA125) has been shown to discriminate between therapy-sensitive and therapy-resistant patients [43]. Extracellular vesicles (EVs) derived from ovarian cancer cells display distinct protein glycosylation patterns that reflect the glycosylation status of the original tumor cells and may serve as potential biomarkers for ovarian cancer. The glycoprotein galectin-3 binding protein (LGALS3BP) was identified in cancer-derived EVs for the first time in ovarian cancer [44, 45]. In a study involving 73 ovarian cancer patients and 70 patients with benign gynecological conditions, elevated serum levels of LGALS3BP and CA125 were observed. When both markers were combined, the sensitivity increased to 86%. LGALS3BP expression is correlated with tumor differentiation grade and recurrent disease during chemotherapy, suggesting the potential use of LGALS3BP and CA125 for ovarian cancer detection and monitoring [46]. In another study, tandem mass spectrometry analysis of the secretome from early-stage 3D ovarian cancer models revealed LGALS3BP as one of the top five candidate biomarkers. This finding was validated in more than 200 primary early-stage ovarian cancer tissues, with LGALS3BP being expressed in 43% of stage I/II tumors and 62% of stage III/IV tumors, indicating a positive association with tumor recurrence [47]. Additionally, a link between LGALS3BP and interferons (IFNs) was established in ovarian cancer. Both IFN-α and IFN-γ were found to increase LGALS3BP mRNA expression and secretion in ovarian carcinoma cell lines, whereas other treatments, such as IL-1β and TNF-α, did not consistently affect LGALS3BP secretion [48].

### O-glycosylation

#### 2.2.1 ***O-GalNAc glycosylation***

Aberrant O-GalNAc glycosylation plays a significant role in various key processes associated with cancer, and changes in O-glycosylation are common [49]. O-glycosylation, a prevalent and diverse posttranslational modification, occurs within the Golgi apparatus and involves a sequential cascade of enzymatic reactions catalyzed by multiple glycosyltransferases. The synthesis of O-glycans begins with the transfer of N-acetylgalactosamine (GalNAc) to serine, threonine or tyrosine residues on proteins, which is mediated by a family of 20 polypeptide GalNAc-transferases (GalNAc-Ts). This initial step results in the production of the Tn antigen (GalNAc-α1-O-Ser/Thr/Tyr), also termed the Thomsen-Nouveau antigen [50]. The Tn antigen subsequently undergoes further branching and capping through subsequent processing steps involving a wide array of distinct glycosyltransferases. The addition of a galactose (Gal) residue to the Tn antigen results in the formation of the T antigen, also known as the Core 1 structure (Galβ1-3-GalNAcα1-O-Ser/Thr) or the Thomsen-Friedenreich (TF) antigen. This enzymatic reaction is carried out by the enzyme T-synthase (core 1 β3-galactosyltransferase). The correct folding of T-Synthase is facilitated by a chaperone called COSMC (Core 1-Specific-Molecular-Chaperone) [51]. In situations where functional T-synthase is absent, the addition of an N-acetylneuraminic acid (Neu5Ac) residue to the GalNAc produces the STn antigen (Neu5Acα2-6GalNAcα-O-Ser/Thr/Tyr), which is catalyzed by the sialyltransferase ST6GalNAc-I [52]. In normal cells, O-glycosylation advances to produce complex O-glycans, which are frequently modified with sialic acid. Elevated global sialylation is strongly associated with cancer and can profoundly affect cell adhesion, cellular recognition, and cell signaling [20]. In ovarian cancer, Tn/STn glycans are detectable, with studies revealing a link between the abnormal expression of GalNAc-Ts and increased tumor aggressiveness [53]. Moreover, the overexpression of *C1GALT1* might interfere with correct T-Synthase folding and activity, thereby contributing to Tn and STn antigen expression (54). Secreted or shed STn antigen-containing O-glycoproteins can enter the bloodstream, providing a straightforward and noninvasive method for the diagnosis and postoperative monitoring of serum tumor markers. The presence of a substantial tumor mass is typically required for this process, making it more common in advanced cancers. Elevated levels of STn (> 38 U/mL) have been identified in the serum of patients with various types of cancer, including ovarian cancer [55, 56]. Additionally, ovarian cancer cells positive for STn are more frequently found at the invasive front of tumors and less commonly in metastatic lesions [57]. Furthermore, Tn and STn expression is associated with a poor prognosis in ovarian cancer patients [58].

#### O-GlcNAc glycosylation

O-GlcNAc glycosylation, also known as O-linked β-N-acetylglucosamine glycosylation, is a type of posttranslational modification in which a single sugar molecule called N-acetylglucosamine (GlcNAc) is attached to serine or threonine residues of cytosolic proteins. This modification is highly dynamic and reversible, with O-GlcNAc transferase (OGT) adding the GlcNAc group and O-GlcNAcase (OGA) removing it. O-GlcNAc glycosylation regulates numerous key cellular processes, including protein stability, signaling, transcriptional regulation, metabolism, cell cycle control, and protein‒protein interactions [59]. In vitro studies have shown that O-GlcNAcylation enhances RhoA/ROCK signaling, resulting in increased migration and invasion of ovarian cancer cells [60], and that changes in O-GlcNAc homeostasis activate the p53 pathway [61]. Furthermore, O-GlcNAcylation of SNAP-23 regulates exosome secretion, whereas downregulation of OGT is positively correlated with exosome release and promotes cisplatin efflux [62]. Recently, a study revealed a link between O-GlcNAc and tumor immune evasion and suggested strategies for improving PD-L1-mediated immune checkpoint blockade therapy by inhibiting O-GlcNAcylation [63].

### Glycosphingolipids

Glycosphingolipids (GSLs) are glycolipids found on the cell surface and are classified into ganglio-, globo-, and lacto-series on the basis of their core structures. Each ganglioside series is linked to specific cell types or tissues and can influence cell adhesion and signaling properties [64]. Within the ganglioside and globo families, certain members exhibit distinct expression patterns in ovarian cancer [65].

#### Gangliosides

Gangliosides are a subclass of GSLs characterized by the presence of one or more sialic acid residues. These molecules are involved in various cellular processes, including cell adhesion and signaling. Gangliosides interact with phospholipids, cholesterol, and transmembrane proteins, playing crucial roles in these cellular functions [64].

##### Disialogangliosides GD2 and GD3

In a retrospective study, the tumor markers gangliosides GD2 and GD3 were evaluated as potential diagnostic biomarkers for ovarian cancer. This study analyzed stored tissue and serum samples from patients diagnosed with invasive epithelial ovarian cancer, as well as samples from healthy donors, individuals with nonmalignant gynecological conditions, and those with other types of cancer. GD2 and GD3 were present in tissues from all stages and subtypes of ovarian cancer according to the FIGO (Fédération Internationale de Gynécologie et d'Obstétrique) classification but were absent in adjacent healthy tissues and other control samples. Furthermore, elevated levels of GD2 and GD3 were detected in the serum of ovarian cancer patients [66]. The diagnostic model based on these gangliosides outperformed the standard biomarker CA125 in diagnosing ovarian cancer, including early-stage (I/II) ovarian cancer [67]. In the overall population (FIGO I–IV), a combination of GD2+ and GD3+ age yielded a sensitivity of 97.6%, surpassing CA125's sensitivity of 63.4% (p < 0.001) while maintaining similar specificity levels (91.2% and 91.8%, respectively). Within the early-stage subset (FIGO I–II), both GD2+, GD3+, age and CA125 exhibited comparable specificity (91.2% and 91.8%, respectively), yet GD2+, GD3+, age achieved a sensitivity of 100%, whereas CA125 demonstrated a lower sensitivity (57.1%) [67].

Exosomes containing GD3, which are isolated from the ascites of human ovarian tumors, were found to cause the arrest of T cell activation when exposed to ganglioside for a short period. However, this exposure did not lead to T cell apoptosis, indicating that prolonged exposure to gangliosides is required to induce T cell death. Additionally, the inhibitory effect of GD3-induced arrest appeared to be dependent on the presence of sialic acid groups, as the enzymatic removal of these groups reversed the inhibitory effect [68].

#### Globo family

The core structure of Galα1-4Galβ1-4Glcβ-Cer is shared by the Globo family of GSLs. Within this family, there are *tumor-associated carbohydrate antigens* (TACAs), such as stage-specific antigen 3 (SSEA-3, also known as GB5), Globo-H (SSEA-3b), and SSEA-4. Among these, Globo-H and SSEA-4 have been extensively studied as potential targets. Both Globo-H and SSEA-4 are derived from SSEA-3, differing only in their terminal moieties, which are fucose and sialic acid, respectively [69]. GSLs, particularly the globo and ganglio series, are associated with and contribute to the transition between epithelial and mesenchymal cells. The dynamic changes in GSL composition provide additional support for the concept of cancer cell plasticity. Moreover, emerging evidence indicates that ganglioside dependent, calcium-mediated mechanisms play a role in maintaining mesenchymal cell characteristics [70]. The GSL P_1_ is an ovarian cancer-associated carbohydrate antigen involved in migration [71].

##### *Globo-H*

The GSL Globo-H is highly expressed in ovarian and other epithelial cancers. Globo-H has been implicated in promoting immunosuppression, angiogenesis, and tumor metastasis [72], and naturally occurring antiglycan antibodies that bind to Globo H-expressing cells can be found in ovarian cancer patients [73]. Developmentally, Globo-H is expressed primarily in undifferentiated embryonic stem cells (ESCs) and disappears upon differentiation [74]. In normal differentiated tissues, its expression is low and is confined mainly to glandular epithelial cells that are inaccessible to the immune system [72]. In contrast, Globo-H is present on the outer membrane of cancer cells, making it an intriguing target for immunotherapies because of its cancer cell specificity.

##### *SSEA-4*

Like Globo-H, stage-specific embryonic antigen-4 (SSEA-4) is a glycosphingolipid (GSL) regulated during development. SSEA-4 is expressed predominantly in early embryonic stages and pluripotent stem cells but is largely absent in differentiated cells and tissues. In cancer cells, SSEA-4 promotes invasion and metastasis by disrupting cell‒cell interactions and inducing a migratory phenotype [69]. Consequently, SSEA-4 appears to be a promising target for anticancer therapy. However, SSEA-4 is expressed in healthy adult tissues, including the ovary [75]. Nevertheless, efforts are underway to establish SSEA-4 as a therapeutic target structure for ovarian carcinoma via CAR T-cell therapy. Remarkable and specific antitumor responses were observed at all doses of CAR T cells used for in vivo efficacy and safety studies conducted on immunodeficient NOD scid gamma (NSG) mice utilizing the high-grade serous ovarian cancer cell line OVCAR4 [76].

### Glycosaminoglycans

In recent years, there has been increasing recognition of the significant roles played by the remodeling of the extracellular matrix (ECM), particularly in terms of altering its mechanical properties, which has spurred extensive investigations within the realm of ovarian cancer research [77]. The organization of the ECM undergoes spatiotemporal regulation, meticulously governing cellular behavior through intricate and synchronized interactions with ECM components. Disruption of the precise regulation of ECM remodeling primarily affects cell fate by modifying rigidity and structure, thereby contributing to the loss of tissue homeostasis. This disruption has been implicated in numerous fundamental characteristics of cancer, including immunosuppression [78, 79], and has shown promise as a diagnostic biomarker for predicting ovarian cancer outcomes [80, 81]. Specifically, chondroitin sulfate disaccharides (CS-Es) are present at elevated levels in the sera of patients with ovarian cancer [82, 83].

Proteoglycans (PGs) and glycosaminoglycans (GAGs) are essential structural and functional components of the extracellular matrix (ECM) and play crucial roles in ovarian cancer [84]. PGs are complex molecules consisting of a protein core covalently linked to GAGs. The biosynthesis of GAGs is a complex, nontemplate-driven process that necessitates the coordinated efforts of various tissue-specific enzymes [85]. In this context, notable glycosaminoglycans (GAGs) include chondroitin sulfate (CS), dermatan sulfate (DS), keratan sulfate (KS), heparan sulfate (HS), and hyaluronic acid (HA) [86], as illustrated in Fig. 1. In ovarian cancer, pericellular HA deposition, regardless of its staining intensity, was significantly associated with malignancy, and in a primary ovarian cancer cohort, it represented an independent unfavorable prognostic marker for overall survival [87]. Notably, in addition to HA, GAGs have the capacity to undergo sulfation, resulting in negatively charged polysaccharide compounds and attachment to a core protein. Changes in the degree and pattern of HS, DS, and CS sulfation are associated with ovarian cancer [88] and its specific cancer-related functions [81, 82, 89, 90]. In ovarian cancer, CS is bound to cell adhesion molecules such as versican and aggrecan, both of which exhibit prognostic value [91, 92]. The classification of PGs is contingent on both the properties of the GAG chains and their expression patterns in human cancer. Dysregulated expression and distribution of PGs and GAGs results in a compromised ECM [93]. Owing to their structural characteristics, PGs represent an interaction platform for various molecular factors to modulate ovarian cancer progression [94]. These effects in cancer may be specific to either individual PGs or the intricate network formed by PGs and multiple ECM factors. Therefore, understanding the mechanisms involving overall or individual PGs/GAGs within the ECM is crucial for understanding the fundamental principles driving ovarian tumor progression [84, 95] and immune evasion [96].

## Glycan-mediated modulation of the immune compartment

Numerous mechanisms elucidating how glycan structures can influence immune responses have been identified. These mechanisms include the creation of a glycoprotein shield on the surface of tumor cells, which hinders immune cells from forming immune synapses; the alteration of interactions with immune cell receptors and glycan-binding proteins (GBPs), which function as scavengers of cytokines or chemokines; and the induction of autoantibodies against glycoproteins displaying aberrant glycosylation. The impact of ovarian cancer cell glycosylation on immunomodulation has been suggested by several studies describing glycosylation-related transcriptome signatures associated with immune cell infiltration and the efficacy of immunotherapies. The 6-gene signature, comprising *ALG8*, *B4GALT5*, *FUT8*, *GCNT2*, *ST6GAL1* and *ST8SIA3*, classifies ovarian cancer patients into high- and low-risk groups, with the latter showing higher levels of immune cell infiltration and response to immune checkpoint inhibition [97]. A risk signature derived from the 6-gene signature through Cox univariate analysis and LASSO Cox regression analysis effectively predicted the overall survival of ovarian cancer patients in both the training and validation cohorts, with P < 0.001 and AUCs > 0.6. Furthermore, the risk score independently predicted the prognosis of ovarian cancer patients. Consistent with these data, four glycosylation-related mRNAs (*ALG8*, *DCTN4*, *DCTN6* and *UBB*) accurately predict prognosis, with high risk values indicating poor prognosis and low immune infiltration [98].

**Fig. 2:**
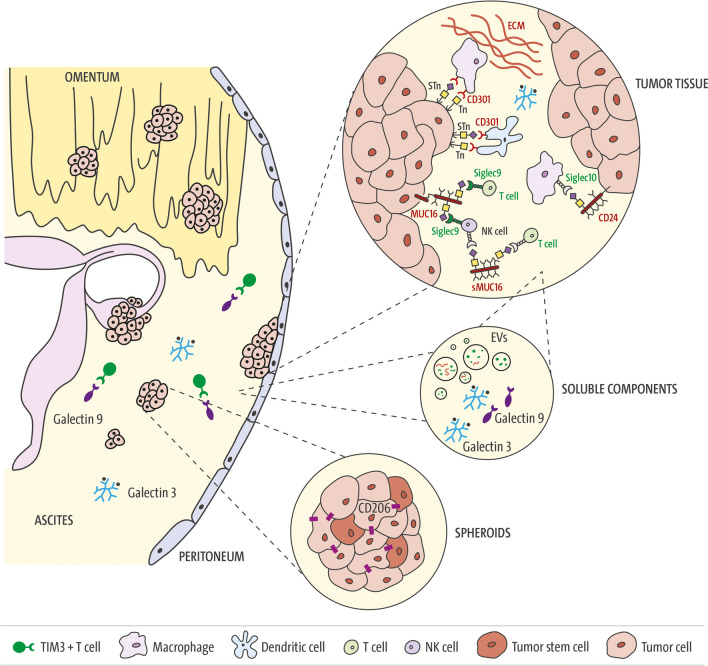
Glycan-related modulation of the immune compartment in ovarian cancer. *Peritoneal cavity*: Ovarian cancer disseminates mainly in the peritoneal cavity, where primary tumors, metastatic lesions and, frequently, the accumulation of malignant ascites can be observed at advanced stages. The ascitic fluid contains single tumor and stroma cells, cell aggregates (spheroids) and soluble components. *Soluble components*: Tumor and stromal cells secrete a great variety of protumorogenic factors, including glycan-binding proteins. Galectin-9 levels in the ascites of ovarian cancer patients are associated with the extent of TIM-3-positive T cells, suggesting that this immune checkpoint axis affects the immunosuppressive environment [93]. Galectin-3 forms oligomers in the extracellular space and leads to the scavenging of glycosylated inflammatory factors, thereby leading to decreased T cell infiltration. In a preclinical study, the combination of a Gal-3 antagonist and PD1/PD-L1 axis blockade significantly reduced ascites accumulation and intraperitoneal metastasis [96]. Ovarian cancer cells release extracellular vesicles (EVs), which exhibit characteristic protein glycosylation signatures from the original tumor cells, such as the glycoprotein galectin-3 binding protein (LGALS3BP) [36, 37]. Furthermore, ascites-derived EVs upregulate Siglec-10 expression in T cells, and cancer-associated adipocyte (CAA) EVs increase tumoral CD24 expression, leading to both the activation of the Siglec-10-CD24 immune axis and the promotion of CD8+ T cell apoptosis [115, 116]. *Spheroids*: Within spheroids, ovarian CSCs are responsible for driving increased expression of the M2 macrophage marker CD206, a C-type lectin that binds high-mannose glycans, implying a more immunosuppressive program in these cellular structures. *Tumor tissue*: Increased O-glycosylation in ovarian cancer cells has been shown to have an immunomodulatory effect. STn and Tn antigens bind to C-type lectin domain family 10 member A (CD301) on type 2 conventional dendritic cells (cDC2s) and macrophages, inhibit DC migration and increase the number of M2-like tumor-associated macrophages [110]. The ECM, which includes a large diversity of proteoglycans and GAGs, directly educates an immunoregulatory macrophage population in ovarian cancer. Siglec-10, which is expressed in tumor-associated macrophages, binds CD24 in a sialylation-dependent manner and leads to immune cells [111]. Sialoglycan-mediated sMUC16/csMUC16-Siglec-9 binding mediates the inhibition of antitumor immune responses [111, 112]

To date, few functional investigations have demonstrated the immunomodulatory effects of particular glycan structures in ovarian cancer. In addition to those mentioned in Sect. "Aberrant glycosylation in ovarian cancer", such as high-mannose, sialyl-Lewis X (SLe^X^), Tn and STn antigens; disialogangliosides GD2 and GD3; the glycolipid Globo-H; and glycosaminoglycans involved in the ECM (HS, DS, CS, and HA), substantial research has indicated the significant involvement of GBPs (Fig. 2). These proteins, also known as glycan-receptors or lectins, bind specific glycan sequences on protein backbones or lipid structures, promoting glycan-code-based cellular processes. Several GBP families are known, three of which play important roles in modulating the immune system: galectins, C-type lectins and Siglecs. Galectins are soluble factors, whereas the majority of C-type lectins and Siglecs are localized in the cell membrane [19, 99]. The differentially expressed glycans in ovarian cancer are juxtaposed with their respective glycan-binding receptors on endothelial or immune cells in Fig. 1 and in the tumor-biological context in Fig. 2.

### Galectins

Galectins are a family of β-galactoside-binding proteins that play a role in tissue repair, adipogenesis, the regulation of immune homeostasis and cancer development. In humans, 15 galectins have been identified [100] and classified into three subtypes according to their protein structure: (I) prototype galectins (galectin 1, 2, 7, 10–15), which contain only one carbohydrate recognition domain (CRD) and exist in a monomer–dimer equilibrium; (II) tandem-repeat-type galectins (e.g., galectins-4, -6, -8 and -9), which comprise two different CRDs in a single polypeptide chain; and (III) chimera-type galectin-3, which is the only family member containing a nonlectin domain linked to a CRD [101]. Galectins are soluble proteins that can be found in the extracellular space, where they bind a variety of glycoconjugates on the cell surface or ECM, as well as in the cell cytoplasm, where they regulate diverse processes through glycan-independent interactions [102].

Galectins are expressed by tumor and immune cells and have been shown to modulate the immune response in cancer through different mechanisms, such as the induction of apoptosis in activated T cells (galectin-1), the promotion of cytokine release into the immunosuppressive environment (galectin-1) or the regulation of T cell exhaustion and, in turn, the modulation of immunotherapy efficacy (galectin-9, Gal-9) [103]. Gal-9 specifically interacts with TIM-3 (HAVCR2) on T cells and induces apoptosis [104, 105]. An interaction between Gal-9, PD-1 and TIM-3 has been recently shown to regulate T cell death as well, thereby representing an attractive target for cancer immunotherapy [106]. In ovarian cancer, the expression of TIM-3 in the ascites of malignant ovarian cancer patients correlates with Gal-9 levels, suggesting an impact of this immune checkpoint axis on the immunosuppressive environment of epithelial ovarian cancer and indicating that TIM-3 is a promising target for immunotherapy [107] (Fig. 2).

The overexpression of galectin-3 (Gal-3) has been described in a variety of cancers and is associated with tumor growth and metastasis. The formation of homodimers and oligomers of Gal-3 in the extracellular space depends on the concentration and availability of ligands. Gal-3 oligomers have the capacity to bind substrates via their CRD, thereby initiating intracellular signal transduction through the clustering of surface proteins and mediating cell‒cell interactions or interactions between cells and the extracellular matrix through the clustering of surface proteins [108, 109]. The latter explains the immunosuppressive role of Gal-3, namely, the accumulation of glycoprotein/Gal-3 lattices in the tissue microenvironment leads to the scavenging of glycosylated soluble factors, e.g., IFN-γ, which in turn decreases CXCL9/10 levels and limits T-cell infiltration (Fig. 2). Low T cell infiltration is a feature of so-called "cold tumors", which include ovarian cancer. In a preclinical study, a Gal-3 antagonist (G3-C12) showed promising effects, especially in combination with a PD-1 (APP) inhibitor, in an ovarian cancer model. G3-C12 was encapsulated in a biodegradable polylactic glycolic acid (PLGA) copolymer, which continuously released the Gal3 antagonist after application. This led to increased T-cell infiltration due to the re-expression of IFN-γ and the activating modulation of dendritic cells (DCs), along with the inhibition of tumor metastasis by intracellular Gal-3. Notably, the combination of G3‒C12 and PD-1/PD-L1 axis blockade with APP significantly reduces ascites accumulation and intraperitoneal metastasis and prolongs mouse survival [110].

### Siglecs

The sialic acid–binding immunoglobulin-like lectin (Siglec) receptor family includes 15 members that are expressed mostly on immune cells. Siglecs contain an amino-terminal V-set immunoglobulin domain that binds sialic acid and a variable number of C2-set immunoglobulin domains. Depending on the signaling domain in the C-terminus, Siglecs may act as immune activators or inhibitors [111]. Hypersialylation of tumor cells has been described for several cancer entities, and blockade of the Siglec-sialoglycan axis has been broadly studied as a potential targeted therapy to reduce aberrant immune responses in both autoinflammatory diseases and cancer [112].

In ovarian cancer, the CD24-Siglec-10 axis has been shown to mediate antitumor immunity and has strong potential for therapeutic intervention. Downregulation of either CD24 or Siglec-10, as well as blockade of the CD24-Siglec-10 axis via monoclonal antibodies, enhanced the phagocytosis of CD24-expressing ovarian cancer cells and resulted in a macrophage-dependent reduction in tumor growth in vivo and increased survival [113]. Siglec-10, which is expressed in tumor-associated macrophages, binds CD24 in a sialylation-dependent manner [114] and activates cytosolic protein tyrosine phosphatases Src-homology 2 domain (SH2)-containing SHP-1/SHP-2-mediated immune cell inhibition [111] (Fig. 2). Two further studies described a dual effect of extracellular vesicles (EVs) on the CD24-Siglec-10 axis: on the one hand, EVs derived from malignant ascites upregulate Siglec-10 expression in T cells, and on the other hand, cancer-associated adipocyte (CAA) EVs deliver SIRT1 to ovarian cancer cells and increase CD24 expression, both of which lead to activation of the abovementioned immune axis and thereby promote CD8+ T cell apoptosis [115, 116].

Siglec-9 (SIGL9) has also been described as a negative glycoimmune checkpoint expressed on myeloid cells, NK cells, and specific T-cell subsets, where it mediates inhibitory effects by binding to sialoglycan ligands on cancer cells. This interaction allows cancer cells to evade immune surveillance [58, 117, 118]. In several tumor types, including ovarian cancer, the upregulation of SIGL9 has been found in tumor-infiltrating leukocytes [119]. In this context, an interaction between SIGL9, which is expressed on T cells and NK cells of ovarian cancer patients, and the soluble form of mucin 16 (sMUC16), a biomarker known as CA125, has been described [58, 120]. Like other mucins, cell surface MUC16 (csMUC16) can also facilitate cell adhesion by interacting with suitable binding partners, such as mesothelin or SIGL9. SIGL9 is an inhibitory receptor that attenuates T cell and NK cell function (Fig. 2). sMUC16/csMUC16-SIGL9 binding likely mediates the inhibition of antitumor immune responses. In an immunotherapeutic approach, Choi et al. developed a SIGL9 antibody that successfully increased antitumor immunity in ovarian cancer. Thus, treatment with the SIGL9 antibody in a humanized mouse model using the ovarian cancer cell line SKOV3, which expresses high levels of SIGL9 ligands and was injected subcutaneously, resulted in reduced tumor growth [121].

### C-type lectins

The large family of C-type lectins (CTLs) are glycan-binding proteins (GBPs) whose function relies on divalent cations, such as Ca^2+^ and Mg^2+^. CTLs are identified by structurally conserved carbohydrate recognition domains (CRDs), which typically contain 110–130 amino acid residues and feature two conserved disulfide bonds. CTLs encompass a diverse range of proteins, including collectins, selectins, endocytic receptors, and proteoglycans. Some of these proteins are secreted, whereas others remain membrane-bound. These CTLs often form oligomers, increasing their affinity for multivalent ligands and enhancing their ability to recognize pattern recognition receptors. One notable feature of CTLs is their significant variability in the types of ligands they can bind with high affinity, including glycans, proteins, lipids, and inorganic compounds [122]. CTLs recognize both pathogens and self-expressed ligands, serving as adhesion molecules, phagocytic receptors, and signaling receptors across diverse biological processes. As essential for maintaining homeostasis, CTLs play crucial roles in innate and adaptive immunity. They are particularly significant in regulating leukocyte and platelet trafficking, as well as tissue remodeling [123]. Interestingly, truncated O-glycans have demonstrated immunomodulatory effects. The Tn antigen is recognized by CD301 (MGL) on conventional type 2 dendritic cells (cDC2s) and macrophages. This interaction inhibits the migration of immature antigen-presenting cells (APCs) and promotes an increase in M2-like tumor-associated macrophages [124] (Fig. 2). In a mouse model of ovarian cancer, the use of glycomimetic peptides to target CD301/MGL was shown to activate dendritic cells (DCs) and immune cells that act against the tumor, leading to enhanced tumor protection, especially when these peptides were used in combination with immune checkpoint inhibitor (ICI) therapy [125, 126]. This finding suggests that the MGL-Tn ligand relationship may hold significant importance in the context of ovarian cancer. Moreover, in the age of ICIs, the recognition of tumor ligands such as tumor glycoantigens, which are created dynamically and interact with immune effector cells during the early stages of tumor development, could be crucial in augmenting the effectiveness of ICIs [127]. Furthermore, CD301/MGL was used to decipher the O-glycoproteome of glycoengineered ovarian cancer cell lines. The identified extracellular proteins possibly interact with CD301/MGL expressed on macrophages and cDC2s, thereby contributing to immune cell modulation [128]. In general, cDC2s play important roles in the immune system during carcinogenesis by presenting tumor antigens to stimulate and activate CD4+ T cells, which are crucial for initiating and regulating immune responses [129]. In cancer, cDC2s are often suppressed by regulatory T cells (Tregs), impairing trafficking to the lymph node and the presentation of tumor-derived antigens to CD4+ T cells [130].

The mannose receptor (MR, CD206) stands out as a unique glycan-binding protein that is expressed in macrophages. While the mannose receptor is just one of numerous glycan-binding proteins, interest has increased in understanding the potential interplay between the macrophage glycome and how it can regulate the activities of related glycan-binding proteins [131]. CD206 is a C-type lectin that binds high-mannose glycans and induces an immunosuppressive program in macrophages. Within ovarian cancer spheroids, CSCs upregulate CD206 in M2 macrophages, inducing an immunosuppressive environment in this cellular compartment [33] (Fig. 2). A recent review and meta-analysis revealed that the presence of tumor-infiltrating CD206-positive macrophages significantly affects oncological outcomes in patients with ovarian cancer and other types of solid tumors [132].

E- and P-selectins are glycoproteins with vital roles in mediating heterophilic cell‒cell interactions in the presence of hydrodynamic flow. The name "selectins" was given to them because of their ability to interact with carbohydrates via their N-terminal CTL domain [133], and their role in pathophysiological processes, including inflammation and metastasis, was recognized [134]. A growing body of evidence indicates that selectins also actively participate in the development of peritoneal carcinomatosis in ovarian cancer [135]. In a recent study by Genduso and colleagues, they investigated how the interplay between integrin β4 (ITGB4) on tumor cells and E-/P-selectin on endothelial cells in the tumor stroma impacts the regulation of tumor growth and influences the immune environment. These findings revealed a highly significant synergy between ITGB4 and E-/P-selectin in controlling tumor growth. This synergy was associated with increased recruitment of CD11b+ Gr-1^Hi^ cells with low granularity, commonly referred to as myeloid-derived suppressor cells (MDSCs), specifically into tumors lacking ITGB4. Tumors lacking ITGB4 exhibit apoptosis and actively attract MDSCs, a cell population well known for its role in promoting tumor growth in various cancer types. This attraction of MDSCs was facilitated by the increased secretion of various chemokines within ITGB4-depleted tumors. The ability of MDSCs to infiltrate tumors was found to be critically dependent on the expression of E-/P-selectin. The analysis of clinical samples supported these findings, indicating an inverse relationship between the expression of ITGB4 in tumors and the number of leukocytes infiltrating the tumor [136].

## Glycan-associated immunotherapeutic approaches

Immunotherapeutic approaches in the context of cancer have gained enormous importance in recent years, achieving remarkable results in some tumor types. Although preclinical studies have shown promising results in patients with ovarian cancer, the efficacy of immunotherapy has been rather moderate, with encouraging clinical results in only a small subgroup of patients [137]. Immune therapeutic strategies, including immune checkpoint blockade (ICB), neoantigen antibodies and vaccines and personalized chimeric antigen receptor (CAR) T-cell therapy, are often based on altered or tumor-specific glycosylation structures or antigens. In the present section, we summarize clinical studies in ovarian cancer that investigated the modulation of glycoimmune complexes for therapeutic purposes.

### *CAR* T-cell therapy

**Fig. 3:**
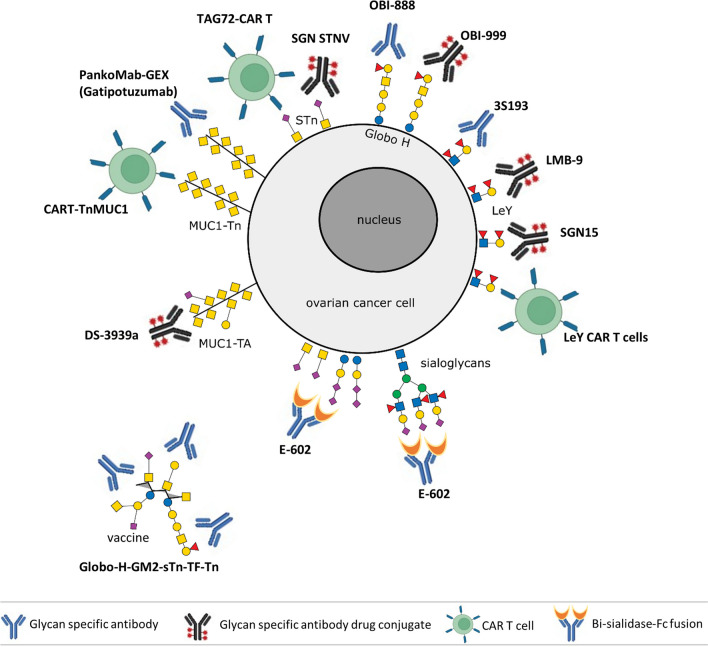
Glycan-associated immunotherapeutic approaches in ovarian cancer. Several preclinical and clinical trials studying the efficacy of glycan-specific antibodies, glycan-specific antibody drug conjugates, glycan-directed CAR T cells or glycan-based vaccines are ongoing or have been conducted. The main glycan structures targeted are STn, Tn, Globo-H, Lewis Y, and sialoglycans. This figure was created via BioRender.com

In ovarian cancer, the application of CAR T-cell therapy still faces some challenges, e.g., the lack of tumor-specific antigens due to the high heterogeneity of this entity, off-target effects, tumor antigen escape or immunosuppression by soluble and/or cellular factors in the tissue microenvironment [138]. Among the tumor antigens that have been investigated in preclinical studies [138] and partly in clinical studies (Table 1, Fig. 3), several highly glycosylated proteins, such as mesothelin, mucin 1, CA125 or CD24, have been identified. However, only a few reports on CAR T cells with specifically altered glycosylation structures have been published thus far. In a syngeneic ovarian cancer mouse model with a genetically modified ID8 cell line, the efficacy of CAR T cells containing a single-chain variable fragment (scFv) from an antibody that recognized a Tn-glycopeptide-antigen was studied. In this cell line (ID8Cosmc-KO), the lack of the transferase-dependent chaperone COSMC (*C1GALT1C1*) led to aberrant O-linked glycosylation and enhanced Tn structures. Tumor regression could be successfully achieved via the use of CARs with high affinity for Tn groups via a single intravenous dose or even more efficiently via intraperitoneal administration. Interestingly, CAR T cells persist for months and allow the treated mice to retard tumor growth in a rechallenge setting [139]. Additionally, CAR T cells targeting TAG72-positive ovarian cancer cells have shown promising results. TAG72, an STn O-glycan carbohydrate found on numerous cell surface glycoproteins [140, 141], is highly expressed in tumor cells, including 90% of epithelial ovarian cancers [142]. The functionality of TAG72-CAR-T cells was demonstrated in an in vivo intraperitoneal mouse model, where strong antigen-dependent cytotoxicity against ovarian cancer cell lines as well as against tumor cells such as patient ascites was observed. Currently, a phase I clinical trial testing the safety, side effects, and best dose of TAG72-CAR T cells in patients with platinum-resistant epithelial ovarian cancer is ongoing (Table 1, Fig. 3). A second phase I clinical trial evaluating the safety, tolerability, feasibility and preliminary efficacy of administering CAR T cells that recognize the tumor antigen TnMUC1 in advanced and treatment-resistant solid cancers, including ovarian cancer, was terminated early owing to unfavorable risk–benefit results (Table 1, Fig. 3). The efficacy of Lewis Y-targeted CAR T cells in advanced solid tumor patients has been evaluated in a clinical trial; however, the outcomes remain unpublished (NCT03851146). Furthermore, ganglioside NGcGM3-targeted CAR-T cells prevent ovarian cancer progression, resulting in low toxicity in healthy tissues [143].

**Table 1:** Selected clinical trials for ovarian cancer with tumor-associated carbohydrate antigen-targeting agents or immune cell therapy. *EAGLE* bispecific fusion protein was developed on the basis of enzyme‒antibody glyco-ligand editing, *ADC* antibody–drug conjugates, ADCC antibody-dependent cell-mediated cytotoxicity, and *CAR-T* chimeric antigen receptor T cells

Type	Modality	Target	Tumor type	Agent / Biological	Function	Phase	Clinical trial number
Antibody	ADCC	Globo-H	advanced or metastatic solid tumors	OBI-888	mAb targeting Globo-H	I/II	NCT03573544
	ADCC	MUC1-Tn	advanced solid tumors	Gatipotuzumab	mAb targeting truncated O-glycans on MUC1	I	NCT03360734
	ADCC	Lewis Y	Ovarian Cancer	3S193	mAb targeting/blocking Lewis Y	II	NCT00617773 NCT01137071
	ADCC	MUC1-Tn	Ovarian Cancer	PankoMab-GEX	mAb targeting truncated O-glycans on MUC1	II	NCT01899599
	ADC	Globo-H	advanced solid tumors	OBI-999	Globo-H mAb and monomethyl auristatin E	I/II	NCT04084366
	ADC	Lewis Y	advanced solid tumors	LMB-9	LMB-9 immunotoxin IV	I	NCT00005858
	ADC	Lewis Y	Ovarian Cancer	SGN15	SGN-15 combined with gemcitabine	II	NCT00051584
	ADC	STn	advanced solid tumors	SGN STNV	mAb targeting STn coupled to monomethyl auristatin E	I	NCT04665921
	ADC	TA-MUC1	advanced solid tumors	DS-3939a	mAb targeting truncated O-glycans on MUC1	I	NCT05875168
	EAGLE	Sialoglycans	advanced solid tumors	E-602	NEU2-Fc for desialylating immunosuppressive sialoglycans	I	NCT05259696
Vaccine	Vaccine	Globo-H-GM2-sTn-TF-Tn	Ovarian Cancer	MabVax/MSKCC	Globo-H-GM2-sTn-TF-Tn-KLH conjugate	I	NCT01248273
	Anti-idiotype	ACA125	Ovarian Cancer	Abagovomab	Functionally imitates the tumor associated antigen CA-125	II /III	NCT00058435/NCT00418574
CAR T	CAR T	STn	Ovarian Cancer	TAG72-CAR T	TAG72-Targeting Chimeric Antigen Receptor T Cells	I	NCT05225363
	CAR T	MUC1-Tn	TnMUC1-Positive Advanced Cancers	CAR T-TnMUC1	MUC1-Tn Targeting Chimeric Antigen Receptor T Cells	I	NCT04025216
	CAR T	Lewis Y	advanced solid tumors	LeY CAR T cells	Lewis Y Targeting Chimeric Antigen Receptor T Cells	I	NCT03851146
Diagnostic	Diagnostic	500 glycoproteoforms	Ovarian Cancer		liquid biopsy—specific glycoproteomic signatures	I	NCT03837327

### Antibodies and antibody‒drug conjugates

There are several ongoing phase I/II clinical studies on the safety and preliminary clinical efficiency of antibodies and antibody‒drug conjugates (ADCs) targeting glycan structures (Table 1, Fig. 3), such as Globo-H (GH), Lewis Y and truncated O-glycans on mucin 1. GH, a hexasaccharide isolated from the human breast cancer cell line MCF-7, is one of the most frequently expressed tumor-associated carbohydrate antigens (TACAs). The efficacy of ADC OB-999, which contains an antibi against GH antibody and the tubulin-targeted cytotoxic payload monomethyl auristatin E [144], is currently being studied in a phase I/II trial (NCT04084366) in patients with advanced solid tumors, including ovarian cancer (Fig. 3).

The antibody–drug conjugate (ADC) SAR566658 is a humanized DS6 antibody that recognizes CA6, a mucin 1 (MUC1)-associated sialoglycotope, linked to the cytotoxic tubulin-binding drug DM4 [145]. CA6 is highly expressed in numerous tumors of epithelial origin, including ovarian cancer [146]. Authors could show an efficient SAR566658 binding to several epithelial carcinoma cell lines in vitro, including one from ovarian origin (OVCA-5), a good ADC internalization, intracellular delivery of DM4 and efficient tumor cell death.

Tumor-associated MUC1 (TA-MUC1) is a tumor-specific transmembrane glycoprotein whose glycosylation is altered due to the deregulation of several sialyltransferases in tumor cells [147]. In a phase I clinical trial (NCT05875168), the safety, tolerability, and efficacy of DS-3939 in patients with advanced solid tumors, including ovarian cancer, will be studied. DS-3939 is a TA-MUC1-directed ADC that includes a number of topoisomerase I inhibitor payloads attached to the antibody via tetrapeptide-based cleavable linkers (Table 1, Fig. 3).

Additionally, gatipotuzumab, a humanized monoclonal antibody recognizing TA-MUC1, was tested in a phase II study (NCT01899599) (Table 1, Fig. 3) for its efficacy and safety as a maintenance therapy in patients with TA-MUC1-positive recurrent ovarian cancer. A total of 216 patients were enrolled and randomized 2:1 to receive gatipotuzumab or placebo every 3 weeks until tumor progression or intolerable toxicity occurred. Although gatipotuzumab was well tolerated, no clinical benefit was observed for gatipotuzumab in comparison to placebo in terms of the secondary efficacy endpoints or in any stratified subgroup [148].

SGN-15 is a chimeric antibody against the Lewis Y antigen, which is conjugated to doxorubicin via an acid-labile, 6-maleimidocaproyl hydrazone linker. Although SGN-15 selectively targeted Lewis Y-expressing cells in preclinical studies [149, 150] (Table 1, Fig. 3), no statistically significant clinical advantage was observed in diverse tumor types, including ovarian cancer (NCT00051584).

A single-arm phase II study tested the efficacy and safety of the humanized anti-Lewis Y monoclonal antibody hu3S193 in diverse cancers, including 29 patients with recurrent epithelial ovarian cancer. The Lewis Y antigen is a difucosylated oligosaccharide carried by numerous glycoproteins and glycolipids. Its overexpression has been described in 75% of ovarian cancers and is associated with patient prognosis. In this study, ovarian cancer patients who achieved a second complete response after platinum-based chemotherapy subsequently received hu3S193 every 2 weeks until disease progression or unacceptable toxicity. Unfortunately, Hu3S193 did not show sufficient clinical activity as a consolidation therapy for these patients [151]. Finally, LMB-9, a disulfide recombinant immunotoxin that directs the cytotoxic potential toward cells expressing the Lewis Y antigen, has been tested in a phase I clinical trial (NCT00005858) (Table 1, Fig. 3); however, the results of this study have not been published thus far.

A further antibody–drug conjugate being investigated in a phase I study (NCT04665921) is SGN STNV. This ADC targets monomethyl auristatin E (MMAE) to tumor cells expressing Sialyl-Thomsen-Nouveau (STn), an O-glycan consisting of a sialic acid residue α2,6-linked to GalNAcα-O-Ser/Thr, which is frequently overexpressed in advanced solid tumors, including ovarian cancer [152]. In preclinical studies, this compound has shown, in addition to its cytotoxic activity, an antitumor response through Fc-mediated effector functions, such as antibody-dependent cellular cytotoxicity (ADCC) and antibody-dependent cellular phagocytosis (ADCP) [153]. The aforementioned phase I study, which started in 2021 and is estimated to enroll 360 patients, will be completed in 2026.

Additionally, the blockade of Siglec-9, a negative immune checkpoint expressed on immune cells, has been evaluated in preclinical studies. Here, a specific Siglec-9 antibody was shown to enhance antitumor functions in vitro and to generate antitumor immunity, leading to a reduced tumor burden in an ovarian cancer mouse model [121].

### Vaccines

CA125 is a highly glycosylated and high-weight cell surface mucin (MUC16) expressed by more than 80% of nonmucinous epithelial ovarian cancers [154]. The level of soluble CA125 is closely associated with disease recurrence and progression [155]. Abagovomab is an anti-idiotic antibody that recognizes CA125 and induces a specific immune response, as confirmed in preclinical and phase I/II studies (NCT00058435)[156, 157]. A significant association between prolonged survival and response to vaccination was previously reported in a preclinical study in which rats were vaccinated with the murine monoclonal anti-idiotypic antibody ACA125 [158]. A phase III trial (NCT00418574) revealed that the administration of abagovomab as maintenance therapy for ovarian cancer patients in first remission does not prolong recurrence-free or overall survival [159]. In this context, Buzzonetti et al. did not find CA125-specific CTL induction by abagovomab. Nevertheless, ovarian cancer patients with CA125-specific CTLs perform better than patients without CA125-specific CTLs do, irrespective of abagovomab treatment [160]. Surprisingly, abagovomab-induced Ab3 (anti-idiotypic antibody) was associated with prolonged recurrence-free survival in patients lacking CA125-specific CTLs [160].

A further vaccination strategy involving synthetic unimolecular pentavalent carbohydrates (Globo-H, GM2, STn, TF, and Tn) on a peptide backbone conjugated to keyhole limpet hemocyanin (KLH) and mixed with the immunological adjuvant QS-21 (NCT01248273) was studied in a phase I trial. Among the 24 ovarian cancer patients enrolled, 20 showed IgG and/or IgM responses to at least three antigens in the vaccine. In the subgroup of patients treated with the highest dose, the individual IgM and/or IgG responses were as follows: Globo-H, 7 (58%); GM2, 4 (33%); STn, 11 (92%); TF, 9 (75%); and Tn, 10 (83%). The advantage of this unimolecular construct is its simple manufacturing process, which allows the addition or replacement of different new antigens [161].

Hypersialylation is a common occurrence in cancer, where sialoglycans are well known for their role in promoting immune evasion by binding to siglecs expressed on immune cells [111]. The overexpression of siglecs presents a challenge when designing therapeutic approaches that target these receptors. To overcome this challenge, the fusion protein E-602 was specifically engineered. E-602 is a fusion protein that incorporates an engineered human sialidase (Neu2), which can cleave terminal sialic acid groups on both immune and tumor cells, and a human IgG1 Fc region. Preclinical studies have shown that E-602-mediated immune activation functions by enhancing antigen-specific priming and T-cell activation as well as by restoring exhausted-like T-cell function. In numerous mouse tumor models, E-602 has shown promising antitumor activity as a monotherapy [162, 163]. Currently, a phase 1/2 study (NCT05259696) in which E-602 is administered alone or in combination with cemiplimab is ongoing for advanced solid tumors. To date, data from a small group of 32 patients with pancreatic or colorectal cancer have been published. Thus, E-602 is well tolerated and leads to dose-dependent desialylation and immune system activation [163].

Another therapeutic strategy that takes advantage of specific glycan‒receptor interactions is the manipulation of macrophages via nanoparticle delivery of siRNAs that can induce immunostimulatory and tumor cytotoxic functions. These nanoparticles have a mannosylated surface designed to specifically target the mannose receptor, which is upregulated in tumor-associated macrophages [164].

## Future models and outlook

In ovarian cancer, the intricate interplay between tumor glycans and immune cell GBPs remains largely uncharted, presenting multifaceted challenges for the scientific community. First, a comprehensive understanding necessitates specialized analytical methods capable of discerning both normal and altered glycan structures or patterns within patients or research models relevant to the field. While the latter have undeniably propelled cancer research forward in recent decades, their limitations have become increasingly evident. In vitro studies often fail to capture the full complexity of biological processes, and fundamental species disparities between humans and mice hinder the seamless translation of research findings into clinical applications [165].

Presently, ongoing investigations into tumor-infiltrating leukocytes and immune cells, in general, rely heavily on single-cell RNA sequencing [166]. However, this technique predominantly captures highly expressed transcripts, leaving a substantial portion of glycosyltransferases and glycan-editing enzymes unaccounted for. This presents yet another formidable challenge: the genotype‒glycotype correlation remains a puzzle yet to be solved. In essence, this implies that the expression of specific genes related to glycosylation fails to provide insights into the actual composition of the glycans found within and on the cell surface.

In summary, multidisciplinary collaboration between basic researchers and medical practitioners is imperative. This collaboration is essential for a comprehensive exploration of the pertinent facets concerning the intricate interplay of glycan structures within various physiological contexts and their interaction with immune cells, all within well-suited experimental systems. Ultimately, this endeavor aims to enhance patient treatment and yield significant survival benefits.

## Data Availability

The data used in this study are derived from public domain resources. References for those sources are available within the article. No original data were generated for this review article.

## References

[1] 2023. Cancer Stat Facts: Ovarian Cancer. In National Cancer Institut - Surveillance, Epidemiology, and End Result Program

[2] Torre LA, Trabert B, DeSantis CE, Miller KD, Samimi G, Runowicz CD, Gaudet MM, Jemal A, Siegel RL (2018) Ovarian cancer statistics, 2018. CA Cancer J Clin 68:284–296 29809280 10.3322/caac.21456 PMC6621554

[3] Cho KR, Shih IM (2009) Ovarian cancer. Annu Rev Pathol 4:287–313 18842102 10.1146/annurev.pathol.4.110807.092246 PMC2679364

[4] Shih Ie M, Kurman RJ (2004) Ovarian tumorigenesis: a proposed model based on morphological and molecular genetic analysis. Am J Pathol 164:1511–1518 15111296 10.1016/s0002-9440(10)63708-x PMC1615664

[5] Singer G, Oldt R 3rd, Cohen Y, Wang BG, Sidransky D, Kurman RJ, Shih IM (2003) Mutations in BRAF and KRAS characterize the development of low-grade ovarian serous carcinoma. J Natl Cancer Inst 95:484–486 12644542 10.1093/jnci/95.6.484

[6] Ahrens TA, M. A., Ibrahim; Berger, Richard; Bielfeld, Alexandra; Boßung, Verena; Bräutigam, Karen; Brucker, Sara Yvonne; Doblinger, Jakob; Dürst, Matthias; Fehm, Tanja; Golic, Michaela; Grewe, Christoph; Griesinger, Georg; Günther, Veronika; Hadji, Peyman; Hagen, Kerstin; Hahnen, Eric; Hanker, Lars; Harbeck, Nadia; Henrich, Wolfgang; Hillemanns, Peter; Hoellen, Friederike; Jäger, Bernadette; Jahnke, Charlotte Marie; Juhasz-Böss, Ingolf; Kienast, Carolin Isabelle; Kiesel, Ludwig; Köhler, Günter; Köster, Frank; Kreis, Nina-Naomi; Krüssel, Jan-Steffen; Kyvernitakis, Ioannis; Liedtke, Cornelia; Louwen, Frank; Ludwig, Michael; Maass, Nicolai; Manz, Maike; Milde-Langosch, Karin; Neubauer, Hans; Oliveira-Ferrer, Leticia; Prieske, Katharina; Rall, Kristin Katharina; Rhiem, Kerstin; Ritter, Andreas; Rody, Achim; Römer, Thomas; Schleußner, Ekkehard; Schmalfeldt, Barbara; Schmutzler, Rita; Schultz, Silke; Solomayer, Erich-Franz; Stubert, Johannes; Stute, Petra; Verlohren, Stefan; Wölber, Linn; Yuan, Juping. 2016. *Molekulare Gynäkologie und Geburtshilfe für die Praxis*: Georg Thieme Verlag KG, Stuttgart

[7] Lheureux S, Braunstein M, Oza AM (2019) Epithelial ovarian cancer: Evolution of management in the era of precision medicine. CA Cancer J Clin 69:280–304 31099893 10.3322/caac.21559

[8] Hwang WT, Adams SF, Tahirovic E, Hagemann IS, Coukos G (2012) Prognostic significance of tumor-infiltrating T cells in ovarian cancer: a meta-analysis. Gynecol Oncol 124:192–198 22040834 10.1016/j.ygyno.2011.09.039 PMC3298445

[9] Chardin L, Leary A (2021) Immunotherapy in Ovarian Cancer: Thinking Beyond PD-1/PD-L1. Front Oncol 11:795547 34966689 10.3389/fonc.2021.795547 PMC8710491

[10] Riviere P, Goodman AM, Okamura R, Barkauskas DA, Whitchurch TJ, Lee S, Khalid N, Collier R, Mareboina M, Frampton GM, Fabrizio D, Sharabi AB, Kato S, Kurzrock R (2020) High Tumor Mutational Burden Correlates with Longer Survival in Immunotherapy-Naive Patients with Diverse Cancers. Mol Cancer Ther 19:2139–2145 32747422 10.1158/1535-7163.MCT-20-0161 PMC7541603

[11] Strickler JH, Hanks BA, Khasraw M (2021) Tumor Mutational Burden as a Predictor of Immunotherapy Response: Is More Always Better? Clin Cancer Res 27:1236–1241 33199494 10.1158/1078-0432.CCR-20-3054 PMC9912042

[12] Nardy AF, Freire-de-Lima L, Freire-de-Lima CG, Morrot A (2016) The Sweet Side of Immune Evasion: Role of Glycans in the Mechanisms of Cancer Progression. Front Oncol 6:54 27014629 10.3389/fonc.2016.00054 PMC4783415

[13] Sperandio M, Gleissner CA, Ley K (2009) Glycosylation in immune cell trafficking. Immunol Rev 230:97–113 19594631 10.1111/j.1600-065X.2009.00795.x PMC2745114

[14] Li X, Xu J, Li M, Zeng X, Wang J, Hu C (2020) Aberrant glycosylation in autoimmune disease. Clin Exp Rheumatol 38:767–775 31694739

[15] Lewis AL, Szymanski CM, Schnaar RL, Aebi M. 2022. Bacterial and Viral Infections. In *Essentials of Glycobiology*, ed. A Varki, RD Cummings, JD Esko, P Stanley, GW Hart, M Aebi, D Mohnen, T Kinoshita, NH Packer, JH Prestegard, RL Schnaar, PH Seeberger, pp. 555–68. Cold Spring Harbor (NY)

[16] Cummings RD, Hokke CH, Haslam SM. 2022. Parasitic Infections. In Essentials* of Glycobiology*, ed. A Varki, RD Cummings, JD Esko, P Stanley, GW Hart, M Aebi, D Mohnen, T Kinoshita, NH Packer, JH Prestegard, RL Schnaar, PH Seeberger, pp. 569–82. Cold Spring Harbor (NY) 35536922

[17] Stowell SR, Ju T, Cummings RD (2015) Protein glycosylation in cancer. Annu Rev Pathol 10:473–510 25621663 10.1146/annurev-pathol-012414-040438 PMC4396820

[18] Thomas D, Rathinavel AK, Radhakrishnan P (2021) Altered glycosylation in cancer: A promising target for biomarkers and therapeutics. Biochim Biophys Acta Rev Cancer 1875:188464 33157161 10.1016/j.bbcan.2020.188464 PMC7855613

[19] RodrIguez E, Schetters STT, van Kooyk Y (2018) The tumour glyco-code as a novel immune checkpoint for immunotherapy. Nat Rev Immunol 18:204–211 29398707 10.1038/nri.2018.3

[20] Pinho SS, Reis CA (2015) Glycosylation in cancer: mechanisms and clinical implications. Nat Rev Cancer 15:540–555 26289314 10.1038/nrc3982

[21] Hirata T, Kizuka Y (2021) N-Glycosylation. Adv Exp Med Biol 1325:3–24 34495528 10.1007/978-3-030-70115-4_1

[22] Reily C, Stewart TJ, Renfrow MB, Novak J (2019) Glycosylation in health and disease. Nat Rev Nephrol 15:346–366 30858582 10.1038/s41581-019-0129-4 PMC6590709

[23] Briggs MT, Condina MR, Klingler-Hoffmann M, Arentz G, Everest-Dass AV, Kaur G, Oehler MK, Packer NH, Hoffmann P (2019) Translating N-Glycan Analytical Applications into Clinical Strategies for Ovarian Cancer. Proteomics Clin Appl 13:e1800099 30367710 10.1002/prca.201800099

[24] Greville G, McCann A, Rudd PM, Saldova R (2016) Epigenetic regulation of glycosylation and the impact on chemo-resistance in breast and ovarian cancer. Epigenetics 11:845–857 27689695 10.1080/15592294.2016.1241932 PMC5193495

[25] Saldova R, Wormald MR, Dwek RA, Rudd PM (2008) Glycosylation changes on serum glycoproteins in ovarian cancer may contribute to disease pathogenesis. Dis Markers 25:219–232 19126966 10.1155/2008/601583 PMC3827796

[26] Biskup K, Braicu EI, Sehouli J, Tauber R, Blanchard V (2014) The serum glycome to discriminate between early-stage epithelial ovarian cancer and benign ovarian diseases. Dis Markers 2014:238197 25183900 10.1155/2014/238197 PMC4145549

[27] Alley WR Jr, Vasseur JA, Goetz JA, Svoboda M, Mann BF, Matei DE, Menning N, Hussein A, Mechref Y, Novotny MV (2012) N-linked glycan structures and their expressions change in the blood sera of ovarian cancer patients. J Proteome Res 11:2282–2300 22304416 10.1021/pr201070k PMC3321095

[28] Biskup K, Braicu EI, Sehouli J, Tauber R, Blanchard V (2017) The ascites N-glycome of epithelial ovarian cancer patients. J Proteomics 157:33–39 28188862 10.1016/j.jprot.2017.02.001

[29] Grzeski M, Taube ET, Braicu EI, Sehouli J, Blanchard V, Klein O (2022) In Situ N-Glycosylation Signatures of Epithelial Ovarian Cancer Tissue as Defined by MALDI Mass Spectrometry Imaging. Cancers (Basel) 14:1021 35205768 10.3390/cancers14041021 PMC8870006

[30] Chen H, Deng Z, Huang C, Wu H, Zhao X, Li Y (2017) Mass spectrometric profiling reveals association of N-glycan patterns with epithelial ovarian cancer progression. Tumour Biol 39:1010428317716249 28681696 10.1177/1010428317716249

[31] Briggs MT, Condina MR, Ho YY, Everest-Dass AV, Mittal P, Kaur G, Oehler MK, Packer NH, Hoffmann P (2019) MALDI Mass Spectrometry Imaging of Early- and Late-Stage Serous Ovarian Cancer Tissue Reveals Stage-Specific N-Glycans. Proteomics 19:e1800482 31364262 10.1002/pmic.201800482

[32] Pan J, Hu Y, Sun S, Chen L, Schnaubelt M, Clark D, Ao M, Zhang Z, Chan D, Qian J, Zhang H (2020) Glycoproteomics-based signatures for tumor subtyping and clinical outcome prediction of high-grade serous ovarian cancer. Nat Commun 11:6139 33262351 10.1038/s41467-020-19976-3 PMC7708455

[33] Raghavan S, Mehta P, Xie Y, Lei YL, Mehta G (2019) Ovarian cancer stem cells and macrophages reciprocally interact through the WNT pathway to promote pro-tumoral and malignant phenotypes in 3D engineered microenvironments. J Immunother Cancer 7:190 31324218 10.1186/s40425-019-0666-1 PMC6642605

[34] Hamester F, Legler K, Wichert B, Kelle N, Eylmann K, Rossberg M, Ding Y, Kurti S, Schmalfeldt B, Milde-Langosch K, Oliveira-Ferrer L (2019) Prognostic relevance of the Golgi mannosidase MAN1A1 in ovarian cancer: impact of N-glycosylation on tumour cell aggregation. Br J Cancer 121:944–953 31659304 10.1038/s41416-019-0607-2 PMC6889143

[35] Machado E, Kandzia S, Carilho R, Altevogt P, Conradt HS, Costa J (2011) N-Glycosylation of total cellular glycoproteins from the human ovarian carcinoma SKOV3 cell line and of recombinantly expressed human erythropoietin. Glycobiology 21:376–386 21030537 10.1093/glycob/cwq170

[36] Zhou Y, Cai X, Wu L, Lin N (2022) Comparative glycoproteomics study on the surface of SKOV3 versus IOSE80 cell lines. Front Chem 10:1010642 36482940 10.3389/fchem.2022.1010642 PMC9723240

[37] Huang YL, Liang CY, Labitzky V, Ritz D, Oliveira T, Cumin C, Estermann M, Lange T, Everest-Dass AV, Jacob F (2021) Site-specific N-glycosylation of integrin alpha2 mediates collagen-dependent cell survival. Iscience 24:103168 34646995 10.1016/j.isci.2021.103168 PMC8501769

[38] Ji Y, Wei S, Hou J, Zhang C, Xue P, Wang J, Chen X, Guo X, Yang F (2017) Integrated proteomic and N-glycoproteomic analyses of doxorubicin sensitive and resistant ovarian cancer cells reveal glycoprotein alteration in protein abundance and glycosylation. Oncotarget 8:13413–13427 28077793 10.18632/oncotarget.14542 PMC5355108

[39] Kratochvilova K, Horak P, Esner M, Soucek K, Pils D, Anees M, Tomasich E, Drafi F, Jurtikova V, Hampl A, Krainer M, Vanhara P (2015) Tumor suppressor candidate 3 (TUSC3) prevents the epithelial-to-mesenchymal transition and inhibits tumor growth by modulating the endoplasmic reticulum stress response in ovarian cancer cells. Int J Cancer 137:1330–1340 25735931 10.1002/ijc.29502

[40] Lin G, Zhao R, Wang Y, Han J, Gu Y, Pan Y, Ren C, Ren S, Xu C (2020) Dynamic analysis of N-glycomic and transcriptomic changes in the development of ovarian cancer cell line A2780 to its three cisplatin-resistant variants. Ann Transl Med 8:289 32355733 10.21037/atm.2020.03.12 PMC7186709

[41] Zahradnikova M, Ihnatova I, Lattova E, Uhrik L, Stuchlikova E, Nenutil R, Valik D, Nalezinska M, Chovanec J, Zdrahal Z, Vojtesek B, Hernychova L, Novotny MV (2021) N-Glycome changes reflecting resistance to platinum-based chemotherapy in ovarian cancer. J Proteomics 230:103964 32898699 10.1016/j.jprot.2020.103964

[42] Zhang X, Wang Y, Qian Y, Wu X, Zhang Z, Liu X, Zhao R, Zhou L, Ruan Y, Xu J, Liu H, Ren S, Xu C, Gu J (2014) Discovery of specific metastasis-related N-glycan alterations in epithelial ovarian cancer based on quantitative glycomics. PLoS ONE 9:e87978 24516574 10.1371/journal.pone.0087978 PMC3916363

[43] Zhao R, Lin G, Wang Y, Qin W, Gao T, Han J, Qin R, Pan Y, Sun J, Ren C, Ren S, Xu C (2020) Use of the serum glycan state to predict ovarian cancer patients’ clinical response to chemotherapy treatment. J Proteomics 223:103752 32209427 10.1016/j.jprot.2020.103752

[44] Escrevente C, Grammel N, Kandzia S, Zeiser J, Tranfield EM, Conradt HS, Costa J (2013) Sialoglycoproteins and N-glycans from secreted exosomes of ovarian carcinoma cells. PLoS ONE 8:e78631 24302979 10.1371/journal.pone.0078631 PMC3840218

[45] Gomes J, Gomes-Alves P, Carvalho SB, Peixoto C, Alves PM, Altevogt P, Costa J (2015) Extracellular Vesicles from Ovarian Carcinoma Cells Display Specific Glycosignatures. Biomolecules 5:1741–1761 26248080 10.3390/biom5031741 PMC4598773

[46] Scambia G, Panici PB, Baiocchi G, Perrone L, Iacobelli S, Mancuso S (1988) Measurement of a monoclonal-antibody-defined antigen (90K) in the sera of patients with ovarian cancer. Anticancer Res 8:761–764 3178164

[47] Lawrenson K, Mhawech-Fauceglia P, Worthington J, Spindler TJ, O’Brien D, Lee JM, Spain G, Sharifian M, Wang G, Darcy KM, Pejovic T, Sowter H, Timms JF, Gayther SA (2015) Identification of novel candidate biomarkers of epithelial ovarian cancer by profiling the secretomes of three-dimensional genetic models of ovarian carcinogenesis. Int J Cancer 137:1806–1817 25204737 10.1002/ijc.29197

[48] Zeimet AG, Stadlmann S, Natoli C, Widschwendter M, Hermann M, Abendstein B, Daxenbichler G, Offner FA, Iacobelli S, Marth C (2000) Peritoneal mesothelial cells as a significant source of ascitic immunostimulatory protein 90K. Anticancer Res 20:4507–4511 11205296

[49] Radhakrishnan P, Dabelsteen S, Madsen FB, Francavilla C, Kopp KL, Steentoft C, Vakhrushev SY, Olsen JV, Hansen L, Bennett EP, Woetmann A, Yin G, Chen L, Song H, Bak M, Hlady RA, Peters SL, Opavsky R, Thode C, Qvortrup K, Schjoldager KT, Clausen H, Hollingsworth MA, Wandall HH (2014) Immature truncated O-glycophenotype of cancer directly induces oncogenic features. Proc Natl Acad Sci U S A 111:E4066–E4075 25118277 10.1073/pnas.1406619111 PMC4191756

[50] Brockhausen I, Wandall HH, Hagen KGT, Stanley P. 2022. O-GalNAc Glycans. In *Essentials of Glycobiology*, ed. A Varki, RD Cummings, JD Esko, P Stanley, GW Hart, M Aebi, D Mohnen, T Kinoshita, NH Packer, JH Prestegard, RL Schnaar, PH Seeberger, pp. 117–28. Cold Spring Harbor (NY)

[51] Ju T, Aryal RP, Kudelka MR, Wang Y, Cummings RD (2014) The Cosmc connection to the Tn antigen in cancer. Cancer Biomark 14:63–81 24643043 10.3233/CBM-130375 PMC5808877

[52] Hugonnet M, Singh P, Haas Q, von Gunten S (2021) The Distinct Roles of Sialyltransferases in Cancer Biology and Onco-Immunology. Front Immunol 12:799861 34975914 10.3389/fimmu.2021.799861 PMC8718907

[53] Sheta R, Bachvarova M, Plante M, Gregoire J, Renaud MC, Sebastianelli A, Popa I, Bachvarov D (2017) Altered expression of different GalNAc-transferases is associated with disease progression and poor prognosis in women with high-grade serous ovarian cancer. Int J Oncol 51:1887–1897 29039611 10.3892/ijo.2017.4147

[54] Chou CH, Huang MJ, Liao YY, Chen CH, Huang MC (2017) C1GALT1 Seems to Promote In Vitro Disease Progression in Ovarian Cancer. Int J Gynecol Cancer 27:863–871 28498248 10.1097/IGC.0000000000000965

[55] Hashiguchi Y, Kasai M, Fukuda T, Ichimura T, Yasui T, Sumi T (2016) Serum Sialyl-Tn (STN) as a Tumor Marker in Patients with Endometrial Cancer. Pathol Oncol Res 22:501–504 26678075 10.1007/s12253-015-0030-9

[56] Kobayashi H, Terao T, Kawashima Y (1992) Sialyl Tn as a prognostic marker in epithelial ovarian cancer. Br J Cancer 66:984–985 1419648 10.1038/bjc.1992.397 PMC1977998

[57] Davidson B, Berner A, Nesland JM, Risberg B, Kristensen GB, Trope CG, Bryne M (2000) Carbohydrate antigen expression in primary tumors, metastatic lesions, and serous effusions from patients diagnosed with epithelial ovarian carcinoma: evidence of up-regulated Tn and Sialyl Tn antigen expression in effusions. Hum Pathol 31:1081–1087 11014575 10.1053/hupa.2000.9776

[58] Belisle JA, Horibata S, Jennifer GA, Petrie S, Kapur A, Andre S, Gabius HJ, Rancourt C, Connor J, Paulson JC, Patankar MS (2010) Identification of Siglec-9 as the receptor for MUC16 on human NK cells, B cells, and monocytes. Mol Cancer 9:118 20497550 10.1186/1476-4598-9-118 PMC2890604

[59] Yang X, Qian K (2017) Protein O-GlcNAcylation: emerging mechanisms and functions. Nat Rev Mol Cell Biol 18:452–465 28488703 10.1038/nrm.2017.22 PMC5667541

[60] Niu Y, Xia Y, Wang J, Shi X (2017) O-GlcNAcylation promotes migration and invasion in human ovarian cancer cells via the RhoA/ROCK/MLC pathway. Mol Med Rep 15:2083–2089 28259907 10.3892/mmr.2017.6244 PMC5364967

[61] de Queiroz RM, Madan R, Chien J, Dias WB, Slawson C (2016) Changes in O-Linked N-Acetylglucosamine (O-GlcNAc) Homeostasis Activate the p53 Pathway in Ovarian Cancer Cells. J Biol Chem 291:18897–18914 27402830 10.1074/jbc.M116.734533 PMC5009264

[62] Qian L, Yang X, Li S, Zhao H, Gao Y, Zhao S, Lv X, Zhang X, Li L, Zhai L, Zhou F, Chen B (2021) Reduced O-GlcNAcylation of SNAP-23 promotes cisplatin resistance by inducing exosome secretion in ovarian cancer. Cell Death Discov 7:112 34001861 10.1038/s41420-021-00489-x PMC8128872

[63] Zhu Q, Wang H, Chai S, Xu L, Lin B, Yi W, Wu L (2023) O-GlcNAcylation promotes tumor immune evasion by inhibiting PD-L1 lysosomal degradation. Proc Natl Acad Sci U S A 120:e2216796120 36943877 10.1073/pnas.2216796120 PMC10068856

[64] Schnaar RL, Sandhoff R, Tiemeyer M, Kinoshita T. 2022. Glycosphingolipids. In *Essentials of Glycobiology*, ed. A Varki, RD Cummings, JD Esko, P Stanley, GW Hart, M Aebi, D Mohnen, T Kinoshita, NH Packer, JH Prestegard, RL Schnaar, PH Seeberger, pp. 129–40. Cold Spring Harbor (NY)

[65] van der Haar Avila I, Windhouwer B, van Vliet SJ (2023) Current state-of-the-art on ganglioside-mediated immune modulation in the tumor microenvironment. Cancer Metastasis Rev 42(3):941–58 37266839 10.1007/s10555-023-10108-z PMC10584724

[66] Webb TJ, Li X, Giuntoli RL 2nd, Lopez PH, Heuser C, Schnaar RL, Tsuji M, Kurts C, Oelke M, Schneck JP (2012) Molecular identification of GD3 as a suppressor of the innate immune response in ovarian cancer. Cancer Res 72:3744–3752 22649190 10.1158/0008-5472.CAN-11-2695 PMC3438513

[67] Galan A, Papaluca A, Nejatie A, Matanes E, Brahimi F, Tong W, Hachim IY, Yasmeen A, Carmona E, Klein KO, Billes S, Dawod AE, Gawande P, Jeter AM, Mes-Masson AM, Greenwood CMT, Gotlieb WH, Saragovi HU (2023) GD2 and GD3 gangliosides as diagnostic biomarkers for all stages and subtypes of epithelial ovarian cancer. Front Oncol 13:1134763 37124505 10.3389/fonc.2023.1134763 PMC10145910

[68] Shenoy GN, Loyall J, Berenson CS, Kelleher RJ Jr, Iyer V, Balu-Iyer SV, Odunsi K, Bankert RB (2018) Sialic Acid-Dependent Inhibition of T Cells by Exosomal Ganglioside GD3 in Ovarian Tumor Microenvironments. J Immunol 201:3750–3758 30446565 10.4049/jimmunol.1801041 PMC6289713

[69] Sigal DS, Hermel DJ, Hsu P, Pearce T (2022) The role of Globo H and SSEA-4 in the development and progression of cancer, and their potential as therapeutic targets. Future Oncol 18:117–134 34734786 10.2217/fon-2021-1110

[70] Cumin C, Huang YL, Rossdam C, Ruoff F, Cespedes SP, Liang CY, Lombardo FC, Coelho R, Rimmer N, Konantz M, Lopez MN, Alam S, Schmidt A, Calabrese D, Fedier A, Vlajnic T, von Itzstein M, Templin M, Buettner FFR, Everest-Dass A, Heinzelmann-Schwarz V, Jacob F (2022) Glycosphingolipids are mediators of cancer plasticity through independent signaling pathways. Cell Rep 40:111181 35977490 10.1016/j.celrep.2022.111181

[71] Jacob F, Anugraham M, Pochechueva T, Tse BW, Alam S, Guertler R, Bovin NV, Fedier A, Hacker NF, Huflejt ME, Packer N, Heinzelmann-Schwarz VA (2014) The glycosphingolipid P(1) is an ovarian cancer-associated carbohydrate antigen involved in migration. Br J Cancer 111:1634–1645 25167227 10.1038/bjc.2014.455 PMC4200095

[72] Berois N, Pittini A, Osinaga E (2022) Targeting Tumor Glycans for Cancer Therapy: Successes, Limitations, and Perspectives. Cancers (Basel) 14(3):645 35158915 10.3390/cancers14030645 PMC8833780

[73] Pochechueva T, Alam S, Schotzau A, Chinarev A, Bovin NV, Hacker NF, Jacob F, Heinzelmann-Schwarz V (2017) Naturally occurring anti-glycan antibodies binding to Globo H-expressing cells identify ovarian cancer patients. J Ovarian Res 10:8 28187738 10.1186/s13048-017-0305-8 PMC5303257

[74] Ho MY, Yu AL, Yu J (2017) Glycosphingolipid dynamics in human embryonic stem cell and cancer: their characterization and biomedical implications. Glycoconj J 34:765–777 27549315 10.1007/s10719-016-9715-x

[75] Virant-Klun I, Skutella T, Hren M, Gruden K, Cvjeticanin B, Vogler A, Sinkovec J (2013) Isolation of small SSEA-4-positive putative stem cells from the ovarian surface epithelium of adult human ovaries by two different methods. Biomed Res Int 2013:690415 23509763 10.1155/2013/690415 PMC3590614

[76] Monzo HJ, Hyytiäinen M, Elbasani E, Kalander K, Wall J, Moyano-Galceran L, Tanjore-Ramanathan J, Jukonen J, Laakkonen P, Ristimäki A, Carlson JW, Lehti K, Salehi S, Puolakkainen P, Haglund C, Seppänen H, Leppä S, Ojala PM (2022) Efficacy and safety of glycosphingolipid SSEA-4 targeting CAR-T cells in an ovarian carcinoma model. Mol Cancer Ther 22(11):1319–31 10.1158/1535-7163.MCT-23-0008 37486980

[77] McKenzie AJ, Hicks SR, Svec KV, Naughton H, Edmunds ZL, Howe AK (2018) The mechanical microenvironment regulates ovarian cancer cell morphology, migration, and spheroid disaggregation. Sci Rep 8:7228 29740072 10.1038/s41598-018-25589-0 PMC5940803

[78] Lecker LSM, Berlato C, Maniati E, Delaine-Smith R, Pearce OMT, Heath O, Nichols SJ, Trevisan C, Novak M, McDermott J, Brenton JD, Cutillas PR, Rajeeve V, Hennino A, Drapkin R, Loessner D, Balkwill FR (2021) TGFBI Production by Macrophages Contributes to an Immunosuppressive Microenvironment in Ovarian Cancer. Cancer Res 81:5706–5719 34561272 10.1158/0008-5472.CAN-21-0536 PMC9397609

[79] Puttock EH, Tyler EJ, Manni M, Maniati E, Butterworth C, Burger Ramos M, Peerani E, Hirani P, Gauthier V, Liu Y, Maniscalco G, Rajeeve V, Cutillas P, Trevisan C, Pozzobon M, Lockley M, Rastrick J, Laubli H, White A, Pearce OMT (2023) Extracellular matrix educates an immunoregulatory tumor macrophage phenotype found in ovarian cancer metastasis. Nat Commun 14:2514 37188691 10.1038/s41467-023-38093-5 PMC10185550

[80] Maeda D, Ota S, Takazawa Y, Aburatani H, Nakagawa S, Yano T, Taketani Y, Kodama T, Fukayama M (2009) Glypican-3 expression in clear cell adenocarcinoma of the ovary. Mod Pathol 22:824–832 19329941 10.1038/modpathol.2009.40

[81] Ten Dam GB, Yamada S, Kobayashi F, Purushothaman A, van de Westerlo EM, Bulten J, Malmstrom A, Sugahara K, Massuger LF, van Kuppevelt TH (2009) Dermatan sulfate domains defined by the novel antibody GD3A12, in normal tissues and ovarian adenocarcinomas. Histochem Cell Biol 132:117–127 19360434 10.1007/s00418-009-0592-2

[82] Biskup K, Stellmach C, Braicu EI, Sehouli J, Blanchard V (2021) Chondroitin Sulfate Disaccharides, a Serum Marker for Primary Serous Epithelial Ovarian Cancer. Diagnostics (Basel) 11(7):1143 34201657 10.3390/diagnostics11071143 PMC8304809

[83] ten Dam GB, van de Westerlo EM, Purushothaman A, Stan RV, Bulten J, Sweep FC, Massuger LF, Sugahara K, van Kuppevelt TH (2007) Antibody GD3G7 selected against embryonic glycosaminoglycans defines chondroitin sulfate-E domains highly up-regulated in ovarian cancer and involved in vascular endothelial growth factor binding. Am J Pathol 171:1324–1333 17717144 10.2353/ajpath.2007.070111 PMC1988881

[84] Wei J, Hu M, Huang K, Lin S, Du H (2020) Roles of Proteoglycans and Glycosaminoglycans in Cancer Development and Progression. Int J Mol Sci 21:5983 32825245 10.3390/ijms21175983 PMC7504257

[85] Raman R, Sasisekharan V, Sasisekharan R (2005) Structural insights into biological roles of protein-glycosaminoglycan interactions. Chem Biol 12:267–277 15797210 10.1016/j.chembiol.2004.11.020

[86] Cho A, Howell VM, Colvin EK (2015) The Extracellular Matrix in Epithelial Ovarian Cancer - A Piece of a Puzzle. Front Oncol 5:245 26579497 10.3389/fonc.2015.00245 PMC4629462

[87] Oliveira-Ferrer L, Schmalfeldt B, Dietl J, Bartmann C, Schumacher U, Sturken C (2022) Ovarian Cancer-Cell Pericellular Hyaluronan Deposition Negatively Impacts Prognosis of Ovarian Cancer Patients. Biomedicines 10:2944 36428513 10.3390/biomedicines10112944 PMC9687866

[88] Liu Y, Chen Y, Momin A, Shaner R, Wang E, Bowen NJ, Matyunina LV, Walker LD, McDonald JF, Sullards MC, Merrill AH Jr (2010) Elevation of sulfatides in ovarian cancer: an integrated transcriptomic and lipidomic analysis including tissue-imaging mass spectrometry. Mol Cancer 9:186 20624317 10.1186/1476-4598-9-186 PMC2913985

[89] Cole CL, Rushton G, Jayson GC, Avizienyte E (2014) Ovarian cancer cell heparan sulfate 6-O-sulfotransferases regulate an angiogenic program induced by heparin-binding epidermal growth factor (EGF)-like growth factor/EGF receptor signaling. J Biol Chem 289:10488–10501 24563483 10.1074/jbc.M113.534263 PMC4036170

[90] Iwahashi N, Ikezaki M, Nishikawa T, Namba N, Ohgita T, Saito H, Ihara Y, Shimanouchi T, Ino K, Uchimura K, Nishitsuji K (2020) Sulfated glycosaminoglycans mediate prion-like behavior of p53 aggregates. Proc Natl Acad Sci U S A 117:33225–33234 33318190 10.1073/pnas.2009931117 PMC7776818

[91] Ghosh S, Albitar L, LeBaron R, Welch WR, Samimi G, Birrer MJ, Berkowitz RS, Mok SC (2010) Up-regulation of stromal versican expression in advanced stage serous ovarian cancer. Gynecol Oncol 119:114–120 20619446 10.1016/j.ygyno.2010.05.029 PMC3000175

[92] Lima MA, Dos Santos L, Turri JA, Nonogaki S, Buim M, Lima JF, de Jesus Viana Pinheiro J, Bueno de Toledo Osorio CA, Soares FA, Freitas VM. 2016 Prognostic value of ADAMTS proteases and their substrates in epithelial ovarian cancer. Pathobiology 83(6):316-26 10.1159/000446244 27359117

[93] Winkler J, Abisoye-Ogunniyan A, Metcalf KJ, Werb Z (2020) Concepts of extracellular matrix remodelling in tumour progression and metastasis. Nat Commun 11:5120 33037194 10.1038/s41467-020-18794-x PMC7547708

[94] Hillemeyer L, Espinoza-Sanchez NA, Greve B, Hassan N, Chelariu-Raicu A, Kiesel L, Gotte M (2022) The Cell Surface Heparan Sulfate Proteoglycan Syndecan-3 Promotes Ovarian Cancer Pathogenesis. Int J Mol Sci 23:5793 35628603 10.3390/ijms23105793 PMC9145288

[95] Vallen MJ, van der Steen SC, van Tilborg AA, Massuger LF, van Kuppevelt TH (2014) Sulfated sugars in the extracellular matrix orchestrate ovarian cancer development: “when sweet turns sour.” Gynecol Oncol 135:371–381 25158037 10.1016/j.ygyno.2014.08.023

[96] Yuan Z, Li Y, Zhang S, Wang X, Dou H, Yu X, Zhang Z, Yang S, Xiao M (2023) Extracellular matrix remodeling in tumor progression and immune escape: from mechanisms to treatments. Mol Cancer 22:48 36906534 10.1186/s12943-023-01744-8 PMC10007858

[97] Xu X, Wu Y, Jia G, Zhu Q, Li D, Xie K (2023) A signature based on glycosyltransferase genes provides a promising tool for the prediction of prognosis and immunotherapy responsiveness in ovarian cancer. J Ovarian Res 16:5 36611197 10.1186/s13048-022-01088-9 PMC9826597

[98] Zhao C, Xiong K, Zhao F, Adam A, Li X (2022) Glycosylation-Related Genes Predict the Prognosis and Immune Fraction of Ovarian Cancer Patients Based on Weighted Gene Coexpression Network Analysis (WGCNA) and Machine Learning. Oxid Med Cell Longev 2022:3665617 35281472 10.1155/2022/3665617 PMC8916863

[99] van Kooyk Y, Rabinovich GA (2008) Protein-glycan interactions in the control of innate and adaptive immune responses. Nat Immunol 9:593–601 18490910 10.1038/ni.f.203

[100] 2024. Gene group: Galectins (LGALS). In *HUGO Gene Nomenclature Committee*: HGNC

[101] Cummings RD, Liu FT, Rabinovich GA, Stowell SR, Vasta GR. 2022. Galectins. In *Essentials of Glycobiology*, ed. A Varki, RD Cummings, JD Esko, P Stanley, GW Hart, M Aebi, D Mohnen, T Kinoshita, NH Packer, JH Prestegard, RL Schnaar, PH Seeberger, pp. 491–504. Cold Spring Harbor (NY) 35536922

[102] Johannes L, Jacob R, Leffler H (2018) Galectins at a glance. J Cell Sci 131(9):208884 10.1242/jcs.208884 29717004

[103] Peixoto A, Miranda A, Santos LL, Ferreira JA (2022) A roadmap for translational cancer glycoimmunology at single cell resolution. J Exp Clin Cancer Res 41:143 35428302 10.1186/s13046-022-02335-z PMC9013178

[104] Yang RY, Rabinovich GA, Liu FT (2008) Galectins: structure, function and therapeutic potential. Expert Rev Mol Med 10:e17 18549522 10.1017/S1462399408000719

[105] Zhu C, Anderson AC, Schubart A, Xiong H, Imitola J, Khoury SJ, Zheng XX, Strom TB, Kuchroo VK (2005) The Tim-3 ligand galectin-9 negatively regulates T helper type 1 immunity. Nat Immunol 6:1245–1252 16286920 10.1038/ni1271

[106] Yang R, Sun L, Li CF, Wang YH, Yao J, Li H, Yan M, Chang WC, Hsu JM, Cha JH, Hsu JL, Chou CW, Sun X, Deng Y, Chou CK, Yu D, Hung MC (2021) Galectin-9 interacts with PD-1 and TIM-3 to regulate T cell death and is a target for cancer immunotherapy. Nat Commun 12:832 33547304 10.1038/s41467-021-21099-2 PMC7864927

[107] Imai Y, Hasegawa K, Matsushita H, Fujieda N, Sato S, Miyagi E, Kakimi K, Fujiwara K (2018) Expression of multiple immune checkpoint molecules on T cells in malignant ascites from epithelial ovarian carcinoma. Oncol Lett 15:6457–6468 29616115 10.3892/ol.2018.8101 PMC5876465

[108] Obermann J, Priglinger CS, Merl-Pham J, Geerlof A, Priglinger S, Gotz M, Hauck SM (2017) Proteome-wide Identification of Glycosylation-dependent Interactors of Galectin-1 and Galectin-3 on Mesenchymal Retinal Pigment Epithelial (RPE) Cells. Mol Cell Proteomics 16:1528–1546 28576849 10.1074/mcp.M116.066381 PMC5546202

[109] Farhad M, Rolig AS, Redmond WL (2018) The role of Galectin-3 in modulating tumor growth and immunosuppression within the tumor microenvironment. Oncoimmunology 7:e1434467 29872573 10.1080/2162402X.2018.1434467 PMC5980349

[110] Li X, Luo L, Jiang M, Zhu C, Shi Y, Zhang J, Qin B, Luo Z, Guo X, Lu Y, Shan X, Liu Y, Du Y, Ling P, You J (2021) Cocktail strategy for “cold” tumors therapy via active recruitment of CD8+ T cells and enhancing their function. J Control Release 334:413–426 33964366 10.1016/j.jconrel.2021.05.002

[111] Crocker PR, Paulson JC, Varki A (2007) Siglecs and their roles in the immune system. Nat Rev Immunol 7:255–266 17380156 10.1038/nri2056

[112] Laubli H, Nalle SC, Maslyar D (2022) Targeting the Siglec-Sialic Acid Immune Axis in Cancer: Current and Future Approaches. Cancer Immunol Res 10:1423–1432 36264237 10.1158/2326-6066.CIR-22-0366 PMC9716255

[113] Barkal AA, Brewer RE, Markovic M, Kowarsky M, Barkal SA, Zaro BW, Krishnan V, Hatakeyama J, Dorigo O, Barkal LJ, Weissman IL (2019) CD24 signalling through macrophage Siglec-10 is a target for cancer immunotherapy. Nature 572:392–396 31367043 10.1038/s41586-019-1456-0 PMC6697206

[114] Chen GY, Tang J, Zheng P, Liu Y (2009) CD24 and Siglec-10 selectively repress tissue damage-induced immune responses. Science 323:1722–1725 19264983 10.1126/science.1168988 PMC2765686

[115] Zheng Q, Du X, Zhang J, Liu Y, Dong W, Dai X, Gu D (2023) Delivery of SIRT1 by cancer-associated adipocyte-derived extracellular vesicles regulates immune response and tumorigenesis of ovarian cancer cells. Clin Transl Oncol 26(1):190–203 37311988 10.1007/s12094-023-03240-3

[116] Li Y, Zhou J, Zhuo Q, Zhang J, Xie J, Han S, Zhao S (2019) Malignant ascite-derived extracellular vesicles inhibit T cell activity by upregulating Siglec-10 expression. Cancer Manag Res 11:7123–7134 31534365 10.2147/CMAR.S210568 PMC6681125

[117] Haas Q, Boligan KF, Jandus C, Schneider C, Simillion C, Stanczak MA, Haubitz M, Seyed Jafari SM, Zippelius A, Baerlocher GM, Laubli H, Hunger RE, Romero P, Simon HU, von Gunten S (2019) Siglec-9 Regulates an Effector Memory CD8(+) T-cell Subset That Congregates in the Melanoma Tumor Microenvironment. Cancer Immunol Res 7:707–718 30988027 10.1158/2326-6066.CIR-18-0505

[118] Jandus C, Boligan KF, Chijioke O, Liu H, Dahlhaus M, Demoulins T, Schneider C, Wehrli M, Hunger RE, Baerlocher GM, Simon HU, Romero P, Munz C, von Gunten S (2014) Interactions between Siglec-7/9 receptors and ligands influence NK cell-dependent tumor immunosurveillance. J Clin Invest 124:1810–1820 24569453 10.1172/JCI65899 PMC3973073

[119] Stanczak MA, Siddiqui SS, Trefny MP, Thommen DS, Boligan KF, von Gunten S, Tzankov A, Tietze L, Lardinois D, Heinzelmann-Schwarz V, von Bergwelt-Baildon M, Zhang W, Lenz HJ, Han Y, Amos CI, Syedbasha M, Egli A, Stenner F, Speiser DE, Varki A, Zippelius A, Laubli H (2018) Self-associated molecular patterns mediate cancer immune evasion by engaging Siglecs on T cells. J Clin Invest 128:4912–4923 30130255 10.1172/JCI120612 PMC6205408

[120] Mitic N, Milutinovic B, Jankovic M (2012) Assessment of sialic acid diversity in cancer- and non-cancer related CA125 antigen using sialic acid-binding Ig-like lectins (Siglecs). Dis Markers 32:187–194 22377735 10.3233/DMA-2011-0872 PMC3826874

[121] Choi H, Ho M, Adeniji OS, Giron L, Bordoloi D, Kulkarni AJ, Puchalt AP, Abdel-Mohsen M, Muthumani K (2021) Development of Siglec-9 Blocking Antibody to Enhance Anti-Tumor Immunity. Front Oncol 11:778989 34869028 10.3389/fonc.2021.778989 PMC8640189

[122] Cummings RD, Chiffoleau E, van Kooyk Y, McEver RP. 2022. C-Type Lectins. In *Essentials of Glycobiology*, ed. A Varki, RD Cummings, JD Esko, P Stanley, GW Hart, M Aebi, D Mohnen, T Kinoshita, NH Packer, JH Prestegard, RL Schnaar, PH Seeberger, pp. 455–74. Cold Spring Harbor (NY)

[123] Brown GD, Willment JA, Whitehead L (2018) C-type lectins in immunity and homeostasis. Nat Rev Immunol 18:374–389 29581532 10.1038/s41577-018-0004-8

[124] Allavena P, Chieppa M, Bianchi G, Solinas G, Fabbri M, Laskarin G, Mantovani A (2010) Engagement of the mannose receptor by tumoral mucins activates an immune suppressive phenotype in human tumor-associated macrophages. Clin Dev Immunol 2010:547179 21331365 10.1155/2010/547179 PMC3038419

[125] Eggink LL, Roby KF, Cote R, Kenneth HJ (2018) An innovative immunotherapeutic strategy for ovarian cancer: CLEC10A and glycomimetic peptides. J Immunother Cancer 6:28 29665849 10.1186/s40425-018-0339-5 PMC5905120

[126] Hoober JK, Eggink LL (2023) Glycomimetic Peptides as Therapeutic Tools. Pharmaceutics 15:688 36840010 10.3390/pharmaceutics15020688 PMC9966187

[127] Mereiter S, Balmana M, Campos D, Gomes J, Reis CA (2019) Glycosylation in the Era of Cancer-Targeted Therapy: Where Are We Heading? Cancer Cell 36:6–16 31287993 10.1016/j.ccell.2019.06.006

[128] Napoletano C, Steentoff C, Battisti F, Ye Z, Rahimi H, Zizzari IG, Dionisi M, Cerbelli B, Tomao F, French D, d’Amati G, Panici PB, Vakhrushev S, Clausen H, Nuti M, Rughetti A (2020) Investigating Patterns of Immune Interaction in Ovarian Cancer: Probing the O-glycoproteome by the Macrophage Galactose-Like C-type Lectin (MGL). Cancers (Basel) 12(10):2841 33019700 10.3390/cancers12102841 PMC7600217

[129] Del Prete A, Salvi V, Soriani A, Laffranchi M, Sozio F, Bosisio D, Sozzani S (2023) Dendritic cell subsets in cancer immunity and tumor antigen sensing. Cell Mol Immunol 20:432–447 36949244 10.1038/s41423-023-00990-6 PMC10203372

[130] Binnewies M, Mujal AM, Pollack JL, Combes AJ, Hardison EA, Barry KC, Tsui J, Ruhland MK, Kersten K, Abushawish MA, Spasic M, Giurintano JP, Chan V, Daud AI, Ha P, Ye CJ, Roberts EW, Krummel MF (2019) Unleashing Type-2 Dendritic Cells to Drive Protective Antitumor CD4(+) T Cell Immunity. Cell 177(556–71):e16 10.1016/j.cell.2019.02.005 PMC6954108 30955881

[131] Cummings RD (2022) The mannose receptor ligands and the macrophage glycome. Curr Opin Struct Biol 75:102394 35617912 10.1016/j.sbi.2022.102394 PMC10243190

[132] Debacker JM, Gondry O, Lahoutte T, Keyaerts M, Huvenne W (2021) The Prognostic Value of CD206 in Solid Malignancies: A Systematic Review and Meta-Analysis. Cancers (Basel) 12(10):2841 10.3390/cancers13143422 PMC8305473 34298638

[133] Bevilacqua M, Butcher E, Furie B, Furie B, Gallatin M, Gimbrone M, Harlan J, Kishimoto K, Lasky L, McEver R et al (1991) Selectins: a family of adhesion receptors. Cell 67:233 1717161 10.1016/0092-8674(91)90174-w

[134] Varki A (1994) Selectin ligands. Proc Natl Acad Sci U S A 91:7390–7397 7519775 10.1073/pnas.91.16.7390 PMC44407

[135] Hassan AA, Artemenko M, Tang MKS, Wong AST (2020) Selectins: An Important Family of Glycan-Binding Cell Adhesion Molecules in Ovarian Cancer. Cancers (Basel) 12:2238 32785160 10.3390/cancers12082238 PMC7463917

[136] Genduso S, Freytag V, Schetler D, Kirchner L, Schiecke A, Maar H, Wicklein D, Gebauer F, Broker K, Sturken C, Milde-Langosch K, Oliveira-Ferrer L, Ricklefs FL, Ewald F, Wolters-Eisfeld G, Riecken K, Unrau L, Krause L, Bohnenberger H, Offermann A, Perner S, Sebens S, Lamszus K, Diehl L, Linder S, Jucker M, Schumacher U, Lange T (2023) Tumor cell integrin beta4 and tumor stroma E-/P-selectin cooperatively regulate tumor growth in vivo. J Hematol Oncol 16:23 36932441 10.1186/s13045-023-01413-9 PMC10022201

[137] Kandalaft LE, Odunsi K, Coukos G (2020) Immune Therapy Opportunities in Ovarian Cancer. Am Soc Clin Oncol Educ Book 40:1–13 32412818 10.1200/EDBK_280539

[138] Zhang XW, Wu YS, Xu TM, Cui MH (2023) CAR-T Cells in the Treatment of Ovarian Cancer: A Promising Cell Therapy. Biomolecules 13:465 36979400 10.3390/biom13030465 PMC10046142

[139] Ranoa DRE, Sharma P, Schane CP, Lewis AN, Valdez E, Marada V, Hager MV, Montgomery W, Wolf SP, Schreiber K, Schreiber H, Bailey K, Fan TM, Hergenrother PJ, Roy EJ, Kranz DM (2023) Single CAR-T cell treatment controls disseminated ovarian cancer in a syngeneic mouse model. J Immunother Cancer 11(5) 10.1136/jitc-2022-006509 PMC10255004 37258040

[140] Shu R, Evtimov VJ, Hammett MV, Nguyen NN, Zhuang J, Hudson PJ, Howard MC, Pupovac A, Trounson AO, Boyd RL (2021) Engineered CAR-T cells targeting TAG-72 and CD47 in ovarian cancer. Mol Ther Oncolytics 20:325–341 33614914 10.1016/j.omto.2021.01.002 PMC7868933

[141] Murad JP, Kozlowska AK, Lee HJ, Ramamurthy M, Chang WC, Yazaki P, Colcher D, Shively J, Cristea M, Forman SJ, Priceman SJ (2018) Effective Targeting of TAG72(+) Peritoneal Ovarian Tumors via Regional Delivery of CAR-Engineered T Cells. Front Immunol 9:2268 30510550 10.3389/fimmu.2018.02268 PMC6254427

[142] Chauhan SC, Vinayek N, Maher DM, Bell MC, Dunham KA, Koch MD, Lio Y, Jaggi M (2007) Combined staining of TAG-72, MUC1, and CA125 improves labeling sensitivity in ovarian cancer: antigens for multi-targeted antibody-guided therapy. J Histochem Cytochem 55:867–875 17478446 10.1369/jhc.7A7213.2007

[143] Cribioli E, Giordano Attianese GMP, Coukos G, Irving M (2022) CAR T cells targeting the ganglioside NGcGM3 control ovarian tumors in the absence of toxicity against healthy tissues. Front Immunol 13:951143 35990626 10.3389/fimmu.2022.951143 PMC9389107

[144] Yang MC, Shia CS, Li WF, Wang CC, Chen IJ, Huang TY, Chen YJ, Chang HW, Lu CH, Wu YC, Wang NH, Lai JS, Yu CD, Lai MT (2021) Preclinical Studies of OBI-999: A Novel Globo H-Targeting Antibody-Drug Conjugate. Mol Cancer Ther 20:1121–1132 33722855 10.1158/1535-7163.MCT-20-0763

[145] Nicolazzi C, Caron A, Tellier A, Trombe M, Pinkas J, Payne G, Carrez C, Guerif S, Maguin M, Baffa R, Fassan M, Adam J, Mangatal-Wade L, Blanc V (2020) An Antibody-Drug Conjugate Targeting MUC1-Associated Carbohydrate CA6 Shows Promising Antitumor Activities. Mol Cancer Ther 19:1660–1669 32451330 10.1158/1535-7163.MCT-19-0826

[146] Kearse KP, Smith NL, Semer DA, Eagles L, Finley JL, Kazmierczak S, Kovacs CJ, Rodriguez AA, Kellogg-Wennerberg AE (2000) Monoclonal antibody DS6 detects a tumor-associated sialoglycotope expressed on human serous ovarian carcinomas. Int J Cancer 88:866–872 11093807 10.1002/1097-0215(20001215)88:6<866::aid-ijc5>3.0.co;2-6

[147] Nath S, Mukherjee P (2014) MUC1: a multifaceted oncoprotein with a key role in cancer progression. Trends Mol Med 20:332–342 24667139 10.1016/j.molmed.2014.02.007 PMC5500204

[148] Ledermann JA, Zurawski B, Raspagliesi F, De Giorgi U, Arranz Arija J, Romeo Marin M, Lisyanskaya A, Poka RL, Markowska J, Cebotaru C, Casado Herraez A, Colombo N, Kutarska E, Hall M, Jacobs A, Ahrens-Fath I, Baumeister H, Zurlo A, Sehouli J (2022) Maintenance therapy of patients with recurrent epithelial ovarian carcinoma with the anti-tumor-associated-mucin-1 antibody gatipotuzumab: results from a double-blind, placebo-controlled, randomized, phase II study. ESMO Open 7:100311 34920291 10.1016/j.esmoop.2021.100311 PMC8685985

[149] Trail PA, Willner D, Lasch SJ, Henderson AJ, Hofstead S, Casazza AM, Firestone RA, Hellstrom I, Hellstrom KE (1993) Cure of xenografted human carcinomas by BR96-doxorubicin immunoconjugates. Science 261:212–215 8327892 10.1126/science.8327892

[150] Willner D, Trail PA, Hofstead SJ, King HD, Lasch SJ, Braslawsky GR, Greenfield RS, Kaneko T, Firestone RA (1993) (6-Maleimidocaproyl)hydrazone of doxorubicin–a new derivative for the preparation of immunoconjugates of doxorubicin. Bioconjug Chem 4:521–527 7508268 10.1021/bc00024a015

[151] Smaletz O, Ismael G, Del Pilar E-D, Nascimento ILO, de Morais ALG, Cunha-Junior GF, Azevedo SJ, Alves VA, Moro AM, Yeda FP, Dos Santos ML, Majumder I, Hoffman EW (2021) Phase II consolidation trial with anti-Lewis-Y monoclonal antibody (hu3S193) in platinum-sensitive ovarian cancer after a second remission. Int J Gynecol Cancer 31:562–568 33664128 10.1136/ijgc-2020-002239

[152] Starbuck K, Al-Alem L, Eavarone DA, Hernandez SF, Bellio C, Prendergast JM, Stein J, Dransfield DT, Zarrella B, Growdon WB, Behrens J, Foster R, Rueda BR (2018) Treatment of ovarian cancer by targeting the tumor stem cell-associated carbohydrate antigen, Sialyl-Thomsen-nouveau. Oncotarget 9:23289–23305 29796189 10.18632/oncotarget.25289 PMC5955411

[153] Schwartz A, Rincon H, Hansen N, Lawrence R, Anderson S, Blesie N, Klussman K, Epp A, Gardai S, Arthur W (2021) Abstract 50: Targeting Sialyl-Thomsen nouveau (STn) antigen with the SGN-STNV antibody-drug conjugate is effective in preclinical studies. Cancer Res 81:5033115805

[154] Hogdall EV, Christensen L, Kjaer SK, Blaakaer J, Kjaerbye-Thygesen A, Gayther S, Jacobs IJ, Hogdall CK (2007) CA125 expression pattern, prognosis and correlation with serum CA125 in ovarian tumor patients. From The Danish “MALOVA” Ovarian Cancer Study. Gynecol Oncol 104:508–515 17113137 10.1016/j.ygyno.2006.09.028

[155] Rustin GJ, Vergote I, Eisenhauer E, Pujade-Lauraine E, Quinn M, Thigpen T, du Bois A, Kristensen G, Jakobsen A, Sagae S, Greven K, Parmar M, Friedlander M, Cervantes A, Vermorken J, Gynecological CI (2011) Definitions for response and progression in ovarian cancer clinical trials incorporating RECIST 1.1 and CA 125 agreed by the Gynecological Cancer Intergroup (GCIG). Int J Gynecol Cancer 21:419–423 21270624 10.1097/IGC.0b013e3182070f17

[156] Pfisterer J, du Bois A, Sehouli J, Loibl S, Reinartz S, Reuss A, Canzler U, Belau A, Jackisch C, Kimmig R, Wollschlaeger K, Heilmann V, Hilpert F (2006) The anti-idiotypic antibody abagovomab in patients with recurrent ovarian cancer. A phase I trial of the AGO-OVAR. Ann Oncol 17:1568–1577 17005631 10.1093/annonc/mdl357

[157] Sabbatini P, Dupont J, Aghajanian C, Derosa F, Poynor E, Anderson S, Hensley M, Livingston P, Iasonos A, Spriggs D, McGuire W, Reinartz S, Schneider S, Grande C, Lele S, Rodabaugh K, Kepner J, Ferrone S, Odunsi K (2006) Phase I study of abagovomab in patients with epithelial ovarian, fallopian tube, or primary peritoneal cancer. Clin Cancer Res 12:5503–5510 17000686 10.1158/1078-0432.CCR-05-2670

[158] Reinartz S, Wagner U, Giffels P, Gruenn U, Schlebusch H, Wallwiener D (2000) Immunological properties of a single-chain fragment of the anti-idiotypic antibody ACA125. Cancer Immunol Immunother 49:186–192 10941901 10.1007/s002620000126 PMC11036980

[159] Sabbatini P, Harter P, Scambia G, Sehouli J, Meier W, Wimberger P, Baumann KH, Kurzeder C, Schmalfeldt B, Cibula D, Bidzinski M, Casado A, Martoni A, Colombo N, Holloway RW, Selvaggi L, Li A, del Campo J, Cwiertka K, Pinter T, Vermorken JB, Pujade-Lauraine E, Scartoni S, Bertolotti M, Simonelli C, Capriati A, Maggi CA, Berek JS, Pfisterer J (2013) Abagovomab as maintenance therapy in patients with epithelial ovarian cancer: a phase III trial of the AGO OVAR, COGI, GINECO, and GEICO–the MIMOSA study. J Clin Oncol 31:1554–1561 23478059 10.1200/JCO.2012.46.4057 PMC5795662

[160] Buzzonetti A, Fossati M, Catzola V, Scambia G, Fattorossi A, Battaglia A (2014) Immunological response induced by abagovomab as a maintenance therapy in patients with epithelial ovarian cancer: relationship with survival-a substudy of the MIMOSA trial. Cancer Immunol Immunother 63:1037–1045 24952307 10.1007/s00262-014-1569-0 PMC11029557

[161] O’Cearbhaill RE, Ragupathi G, Zhu J, Wan Q, Mironov S, Yang G, Spassova MK, Iasonos A, Kravetz S, Ouerfelli O, Spriggs DR, Danishefsky SJ, Sabbatini PJ (2016) A Phase I Study of Unimolecular Pentavalent (Globo-H-GM2-sTn-TF-Tn) Immunization of Patients with Epithelial Ovarian, Fallopian Tube, or Peritoneal Cancer in First Remission. Cancers (Basel) 8:46 27110823 10.3390/cancers8040046 PMC4846855

[162] Peng L, Cao L, Nerle S, LeBlanc R, Das A, Shelke S, Turner A, Che J, Siddiquee Z, Xu H, Xu L, Gatlin W, Broderick J (2021) 843 Development and engineering of human sialidase for degradation of immunosuppressive sialoglycans to treat cancer. J ImmunoTher Cancer 9:A884

[163] Luke JJ, Johnson M, Tolcher A, Chen CT, Dai T, Curti BD, El-Khoueiry A, Sznol M, Henick BS, Horak C, Jayaraman P, Cole CB, Wilson D, Cao L, Peng L, Feltquate D, Lathers D, Sharma MR (2023) Abstract CT034: GLIMMER-01: initial results from a phase 1 dose escalation trial of a first-in-class bi-sialidase (E-602) in solid tumors. Cancer Res 83:CT034

[164] Ortega RA, Barham W, Sharman K, Tikhomirov O, Giorgio TD, Yull FE (2016) Manipulating the NF-kappaB pathway in macrophages using mannosylated, siRNA-delivering nanoparticles can induce immunostimulatory and tumor cytotoxic functions. Int J Nanomedicine 11:2163–2177 27274241 10.2147/IJN.S93483 PMC4876941

[165] Leenaars CHC, Kouwenaar C, Stafleu FR, Bleich A, Ritskes-Hoitinga M, De Vries RBM, Meijboom FLB (2019) Animal to human translation: a systematic scoping review of reported concordance rates. J Transl Med 17:223 31307492 10.1186/s12967-019-1976-2 PMC6631915

[166] Guruprasad P, Lee YG, Kim KH, Ruella M (2021) The current landscape of single-cell transcriptomics for cancer immunotherapy. J Exp Med 218:e20201574 33601414 10.1084/jem.20201574 PMC7754680

[167] Mehta AY, Cummings RD (2020) GlycoGlyph: a glycan visualizing, drawing and naming application. Bioinformatics 36:3613–3614 32170934 10.1093/bioinformatics/btaa190 PMC7267839

